# Spatial Organization and Recruitment of Non-Specific T Cells May Limit T Cell-Macrophage Interactions Within *Mycobacterium tuberculosis* Granulomas

**DOI:** 10.3389/fimmu.2020.613638

**Published:** 2021-01-20

**Authors:** Jess A. Millar, J. Russell Butler, Stephanie Evans, Nicole L. Grant, Joshua T. Mattila, Jennifer J. Linderman, JoAnne L. Flynn, Denise E. Kirschner

**Affiliations:** ^1^ Department of Epidemiology, University of Michigan, Ann Arbor, MI, United States; ^2^ Department of Computational Medicine and Bioinformatics, University of Michigan, Ann Arbor, MI, United States; ^3^ Department of Health and Biomedical Sciences, AdventHealth University, Orlando, FL, United States; ^4^ Department of Microbiology and Immunology, University of Michigan Medical School, Ann Arbor, MI, United States; ^5^ Department of Infectious Diseases and Microbiology, University of Pittsburgh, Pittsburgh, PA, United States; ^6^ Department of Chemical Engineering, University of Michigan, Ann Arbor, MI, United States; ^7^ Department of Microbiology and Molecular Genetics and the Center for Vaccine Research, University of Pittsburgh, Pittsburgh, PA, United States

**Keywords:** T cell, macrophage, *Mycobacterium tuberculosis*, lung, computational model, granuloma

## Abstract

Tuberculosis (TB) is a worldwide health problem; successful interventions such as vaccines and treatment require a 2better understanding of the immune response to infection with *Mycobacterium tuberculosis* (Mtb). In many infectious diseases, pathogen-specific T cells that are recruited to infection sites are highly responsive and clear infection. Yet in the case of infection with Mtb, most individuals are unable to clear infection leading to either an asymptomatically controlled latent infection (the majority) or active disease (roughly 5%–10% of infections). The hallmark of Mtb infection is the recruitment of immune cells to lungs leading to development of multiple lung granulomas. Non-human primate models of TB indicate that on average <10% of T cells within granulomas are Mtb-responsive in terms of cytokine production. The reason for this reduced responsiveness is unknown and it may be at the core of why humans typically are unable to clear Mtb infection. There are a number of hypotheses as to why this reduced responsiveness may occur, including T cell exhaustion, direct downregulation of antigen presentation by Mtb within infected macrophages, the spatial organization of the granuloma itself, and/or recruitment of non-Mtb-specific T cells to lungs. We use a systems biology approach pairing data and modeling to dissect three of these hypotheses. We find that the structural organization of granulomas as well as recruitment of non-specific T cells likely contribute to reduced responsiveness.

## Introduction

Tuberculosis (TB) is caused by infection with *Mycobacterium tuberculosis* (Mtb). It is one of the leading causes of death due to infectious disease, killing 1.7 million people per year (1). The pathologic hallmark of this infection is the formation of lung granulomas, which are collections of host immune cells (e.g. macrophages & T lymphocytes) that organize in an attempt to contain and eliminate the infection (2–4). Although bacterial infection preferentially occurs within macrophages, T cells are key players in the proper functioning of granulomas, and are necessary for macrophage activation (2, 5–7).

T cells play a central role in the host adaptive immune response. CD4+ T cells are activated by binding MHC class II (MHCII) complexes on the surface of antigen presenting cells like macrophages. CD4+ T cells provide help for CD8+ T cells and once activated, both CD4+ and CD8+ T cells serve a number of immune roles such as cytotoxic function, regulatory function, and cytokine production, (e.g. interferon-gamma (IFN-γ) and TNF) that recruit other immune cells and activate macrophages (8–11). Activated macrophages kill Mtb and also produce cytokines and chemokines that recruit other immune cells (2, 12, 13). Mtb-specific T cells play an important role in controlling Mtb infection by influencing the initiation and maintenance of the adaptive immune response, leading to formation of lung granulomas (14, 15). T cells have been shown to be necessary for control of Mtb infection in studies in non-human primates (NHPs) and mice (16–20), and also from studies from humans who are co-infected with HIV-1 and do much worse. Since granulomas are the infection sites within lungs and provide the potential for frequent interactions between Mtb and host immune cells, we expect them to be enriched in Mtb-responsive T cells (i.e. producing cytokines in response to Mtb). Surprisingly, it has been observed that in granulomas from non-human primates, on average <10% of T cells are producing canonical T cell cytokines (IFN-γ, TNF, IL-2, IL-17, or IL-10) throughout the course of Mtb infection (21). This low level of cytokine-producing T cells could be one explanation for how granulomas balance excessive inflammation with bacterial control. Regardless, since 2 billion people in the world are infected with TB, it is useful to understand this delicate balance of T-cell responsiveness and why the frequencies of cytokine-producing T cells in granulomas are lower than expected.

There are a few lines of thinking that have been explored to date to explain these observed low levels of Mtb-responsive T cells observed during infection. One hypothesis is that T cells may become exhausted during Mtb infection, as exhausted T cells have been described in other chronic infectious diseases (22–25). However, we have shown through both experimental and computational work that T cell exhaustion is limited in most NHP TB granulomas (26). A second hypothesis is that T cells are down-regulated directly by the action of Mtb. Mtb’s role in regulating parts of the immune system has been established in studies involving Mtb-derived glycolipids inhibiting pathways in antigen presentation (27–31). Downstream, this would lead to reduced stimulation of T cells. A third hypothesis is that the spatial organization of granulomas affects the ability of T cells to reach macrophages and thus be activated *via* antigen presentation (32–34). The structural organization of granulomas tends toward a typical pattern: Mtb are mostly found within the caseous necrotic core or in epithelioid macrophages adjacent to the core of granulomas, which is then surrounded by layers of macrophages and lymphocytes (35). We provided evidence that T cells had a higher likelihood of exhaustion after penetrating deeper into the granuloma where they could encounter Mtb antigen, but this penetration of T cells occurs infrequently in established granulomas (26). Compounding T cell-macrophage interaction dynamics is the recruitment of T cells into granulomas. T cells localize to and are rapidly recruited into mycobacterial granulomas in the absence of antigen recognition (36–38). If the majority of T cells recruited are Mtb non-specific, Mtb-specific T cells would be less likely to find macrophages and become fully activated due to crowding. Thus, a fourth hypothesis is that non-specific T cells are recruited to granulomas. During chronic infections, there are ongoing signals that can recruit non-specific T cells into lungs due to both the inflammatory nature of granulomas and also the highly vascularized lung environment. In addition, it has also been shown that 90% of non-Mtb-specific T cells are lung tissue resident memory T cells (39, 40). Here we test hypotheses to determine the potential contribution of Mtb modulation, granuloma spatial organization, and T cell recruitment. Our goal is to determine how these factors contribute, either alone or together, to the relatively low levels of observed cytokine-producing T cells established within granulomas during Mtb-infected NHPs.

To address these studies, we need an approach that can explore and compare these hypotheses. The spatial organization of granulomas is crucial to outcomes, as has been suggested in NHPs, mice and rabbit studies (36, 41, 42). In addition, temporal dynamics are important, tracking discrete cells and bacteria as they evolve over the course of granuloma formation and maintenance. Finally, events that participate in the immune response to Mtb occur over biological scales ranging from molecules to cells to tissue. Thus, our approach must accommodate all of these features. To this end, we use a systems biology approach, pairing computational multi-scale modeling with experimental studies in NHPs. Our lab has previously created a multi-scale (intracellular through tissue scales) agent-based model (ABM), *GranSim*, that tracks bacteria and individual immune cells as agents (26, 32, 33, 43–45). This model captures the host response to Mtb and allows spatial tracking of granuloma formation and function. It also tracks bacterial heterogeneity in terms of growth and division by following each individual bacterium within its micro-environments (intracellular, extracellular and trapped within caseum) over time. Using an agent-based model has the additional advantage that it can capture emergent behavior (in this case, the formation of the granuloma) through rules governing immune cell interaction. Herein, we modify *GranSim* to include an additional sub-model that tracks intracellular-level dynamics of macrophage antigen presentation to examine the impact of Mtb on antigen presentation and thus to T cell outcomes within granulomas. To do this, we integrate our previously published model of Mtb-mediated down-regulation of MHCII presentation of peptides (46, 47) within each macrophage in *GranSim*. This will allow us to explore mechanisms of Mtb downregulation of antigen presentation on T-cell responsiveness. At the same time, this multi-scale model can aid understanding of how granuloma structure impacts macrophage and T cell dynamics and also how recruitment to lung granulomas balances T cell specificity/non-specificity. We pair our modeling studies with datasets from NHP granulomas to calibrate and validate our models and predictions.

## Methods

### Immunohistochemistry and Imaging

**Figure 1:**
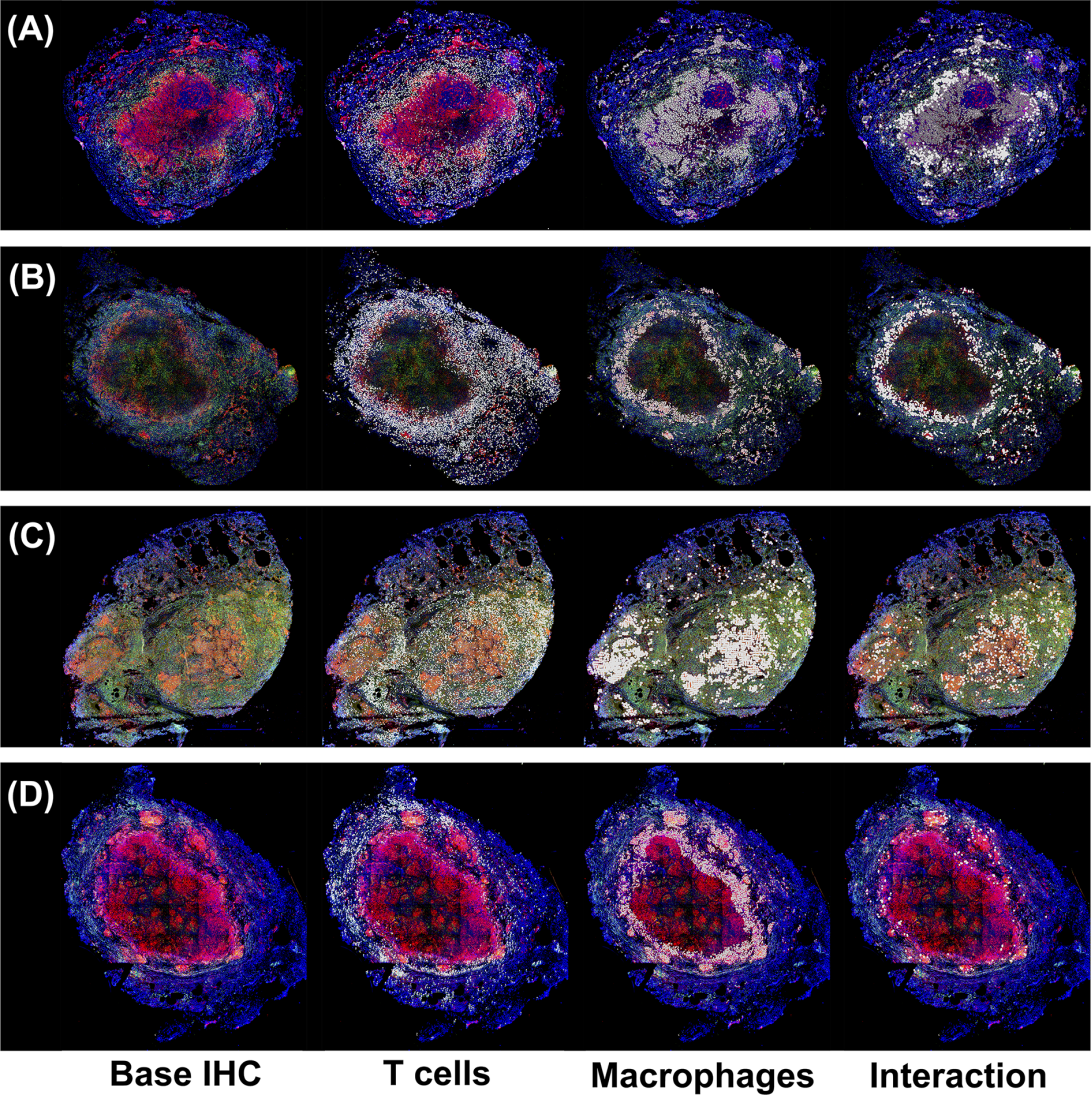
Immunohistochemistry analysis of four non-human primates (NHP) granulomas [shown in Panels (**A-D**)] examining spatial distributions of both T cells and macrophages, and also where they intersect. Four distinct, randomly chosen granuloma images with extracted cell distributions. Column 1 shows the immunohistochemically stained preparation for CD3 (green), CD11c+ macrophages (red), and nuclei (dark blue). White points represent Geographical Information Systems Technology (GIS) analyses of these images revealing cell locations for T cells (Column 2), macrophages (Column 3), and their intersections (Column 4), as follows. Rows represent four distinct granulomas. The data for the cell numbers in these granulomas are given in **Table 2**. On average, about 9.75% (median 8.6%, StDev is 4.5%) of T cells interacted with macrophages.

Four randomly selected, formalin fixed paraffin embedded (FFPE) granulomas were derived from 3 cynomolgus macaques (Macaca fascicularis), necropsied at approx. 10-11 weeks post infection (**Figures 1A–D**), and were deparaffinized and antigen retrieval was performed as previously indicated (35). Granulomas were stained with cocktails of antibodies including polyclonal rabbit anti-CD3 (Agilent Technologies, Santa Clara, CA), IgG2a mouse anti-CD11c (clone 5D11; Leica Microsystems, Buffalo Grove, IL). Primary antibodies were labeled with fluorochrome-labeled secondaries including anti-isotype (IgG2a) specific antibodies (Jackson ImmunoResearch, West Grove, PA). Coverslips were mounted with Prolong Gold with DAPI (ThermoFisher Scientific) and the sections were imaged on an Olympus FluoView confocal microscope (Center Valley, PA) or Nikon e1000 epifluorescence microscope (Nikon Instruments, Melville, NY) with Nikon NIS Elements (Nikon Instruments).

### Geographical Information Systems to Extract T cell-Macrophage Interactions in a Granuloma

Granulomas were obtained and stained as described above. DAPI stained images were provided together with the IHC. We applied an unsupervised classification, iso-cluster, image-classification process to four randomly selected, original immunohistochemically, stained NHP granuloma digital microscopy images. This initial classification technique generated between 28 and 45 classifications. Classes correspond to different cell types, even portions of cells like cell borders, caseum, cellular debris, sample background, and co-expression. These initial classifications were collapsed to the single cell types of interest. For accuracy assessment, the classified image was superimposed onto the DAPI image to ensure that cell location and size were correct. In addition, this process removed cellular debris versus true cells. These classified raster images, where the objects in the image are defined by individual pixels instead of vectors, were then converted to vector-based or polygons and the polygons were then assessed for classification errors. The two different polygon images were subjected to a join technique that recorded the locations where different polygons intersected. From the classification process we created a raster, classified image. This raster image was converted to a vector (polygons) image (ArcPro 2.6). We extracted individual cell distributions by cell type (T cells and macrophages only). We then performed a spatial join (ArcPro 2.6) between the T cell/macrophage distributions based on cell-cell interactions to determine overlap and/or border interactions. The cells were marked on the images, and the numbers of each type together with the interactions were quantified.

### Multi-Scale Model Overview

**Figure 2:**
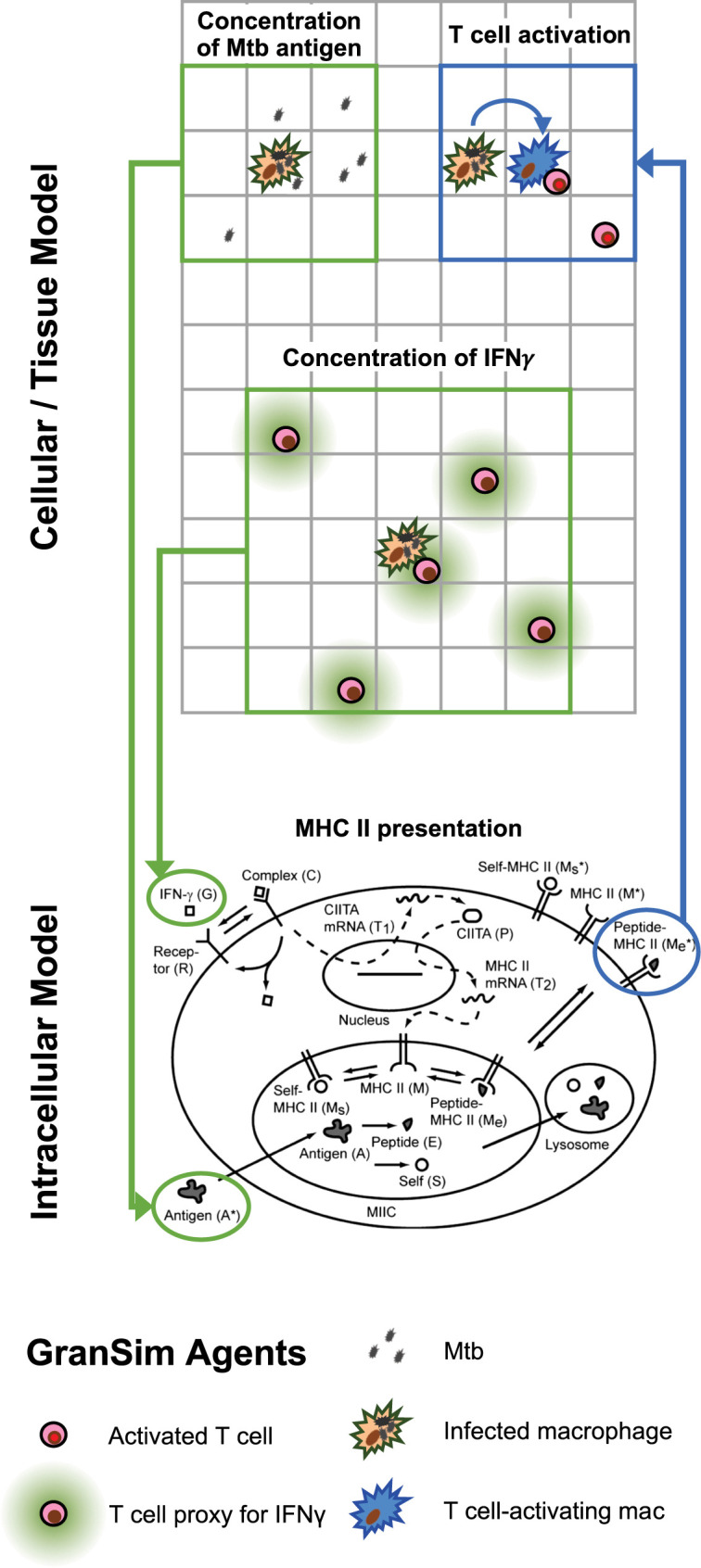
Multi-scale model schematic showing the integration of the intracellular scale model into the mesoscale cellular/tissue model. Within our combined multi-scale model, the cellular/tissue scale is modeled with *GranSim*, an agent-based model capturing dynamics of immune cells, effector molecules and mycobacteria within lungs leading to formation of granulomas. Full cellular and molecule dynamics are not shown for *GranSim*, only the places where the intracellular model links with *GranSim*. For the intracellular model, 14 non-linear ordinary differential equations (ODEs) are represented by this schematic for 14 variables (published previously and listed in **Table S1** for reference here). *GranSim* linking is accomplished *via* inputs to ODEs (green arrows) including the concentration of free IFN-γ (calculated based on T cell numbers) and concentration of free Mtb antigen (calculated using the number of Mtb in a macrophage’s one Moore neighborhood). The ODE output of MHCII-Mtb complexes on a macrophage surface is linked back into *GranSim* (blue arrow). Once these macrophages reach a threshold of surface MHCII-Mtb complexes, they are able to activate T cells within their neighborhood.

To test our three hypotheses and address how bacterial factors, granuloma spatial organization and T cell recruitment lead to reduced T-cell responsiveness, we create a next-generation computational model. Briefly, the main model (mesoscale) operates at the cellular/tissue scales, tracking host immune cells and individual Mtb in the immune system environment, leading to granuloma formation. *GranSim* is an agent-based model (ABM) drawing on well-described cellular and pro- and anti- inflammatory cytokine interactions that is continuously updated and curated with the latest data. These dynamics are all captured between immune cells and individual Mtb using stochastic simulations, operating in two dimensions (2D) [with versions working in three dimensions, but not used here (48)]. We now link an intracellular scale sub-model, capturing MHCII processing and presentation by macrophages using a system of ordinary differential equations (ODEs) previously described (46). The cellular/tissue scales and intracellular scale sub-model are linked through the processes of IFN-γ receptor ligand binding, Mtb antigens, and MHCII Mtb-peptide complexes on macrophage surfaces to activate T cells (**Figure 2**).

### Cellular/Tissue Scale Model

#### Hybrid Multi-Scale Model (*GranSim*)

In this work we build an antigen presentation model into our existing hybrid multi-scale agent-based model (ABM) of granuloma formation, *GranSim*. *GranSim* has been curated and used for testing hypotheses in TB since 2004. The model has been developed in conjunction with extensive experimental datasets regarding the immune response to *M. tuberculosis* within the lungs of non-human primates (NHP), leading to the formation of granulomas (32, 33) (see our detailed website http://malthus.micro.med.umich.edu/GranSim for full model details, all published manuscripts using this model and an executable program). *GranSim* tracks the cellular immune response in lungs following infection with *Mtb* that ultimately leads to emergence of a granuloma (if the initial infection is not cleared). *GranSim* is an agent-based (individual-based) model that is comprised of five features:

#### Agents

Immune cells that are individually tracked as follows: four macrophage states (resting, activated, infected, and chronically infected- see below for more details), three T cell classes (cytotoxic, IFNγ producing, and regulatory), and cytokines and chemokines IFN-γ, TGF, IL10, TNF, CCL2, CXCL9, and CCL5. In addition, individual bacterium are each tracked and are in one of three environments leading to different growth states (intracellular, extracellular and non-replicating trapped in caseum).

#### The Environment

The model environment represents a section of lung tissue that is 4 mm x 4 mm in size, allowing for granulomas to grow to a size that is average of what is observed *in vivo.* The grid is 2D (although we have a 3D version available- see http://malthus.micro.med.umich.edu/3D-GranSim/) and the model grid is subdivided into 20 micron x 20 micron microcompartments. 20 microns is the average size of our largest immune cell class, macrophages. The lung grid also is populated with blood vessels that are placed on the grade based on NHP studies of healthy lungs. These portals are where cells, chemokines, or cytokines can enter the lung space.

#### Rules

Rules are based on probabilistic interactions between cells and the lung environment, derived and validated on extensive datasets of observed interactions of NHP immune cells and molecules. The list of rules is extensive and it is housed on our *GranSim* website.

#### Parameters

*GranSim* is parameterized by dozens of parameters that have been estimated on datasets over the past 15 years. Further, we study their values and impacts using both uncertainty and sensitivity analyses. The last piece of an ABM is to define the time steps of the fastest process occurring on the grid. Here, that process is molecular diffusion, which is on a time scale of 6 s. When simulated, *GranSim* computes rules and agents at the cell and molecular scale and leads to emergent behavior of a granuloma that reads out at the tissue scale. For an executable file and detailed model rules, please see our website which is continuously curated on a regular basis http://malthus.micro.med.umich.edu/GranSim.

### Intracellular MHCII Presentation Model

Antigen presentation occurs when an antigen presenting cell presents foreign antigenic peptides to T cells to activate an adaptive immune response. This process of antigen presentation occurs within lymph nodes on a continual basis and in other locations during infection. Within granulomas, activation of CD4+ T helper cells depends on presentation of Mtb derived peptide–MHC class II complexes (pMHCII) presented on the surface of macrophages (13, 49). T cell activation is required for granulomas to control infection, as it induces IFN-γ secretion, which, in conjunction with other factors, activates macrophages to kill Mtb (13). This is the key step in Mtb cell-mediated immunity and may help determine the outcome of infection (49). In baseline *GranSim*, T cells are recruited to the site of the infection as already activated, with their specificity based on parameter probabilities. This tended to overshoot the proportion of activated T cells as we identified previously in **Figure S1** (21).

Previously, we created a model that describes MHC class II-mediated antigen presentation by antigen-presenting cells (46). This model is developed as an intracellular scale model representing a single antigen presenting cell (e.g. a macrophage). The model comprises 14 ODEs and was created in the context of capturing datasets from multiple *in vitro* studies. The model includes all of the intracellular events occurring during the process of antigen presentation: INFγ-receptor ligand binding, leading to MHC class II transcription through CIITA and uptake of Mtb antigens and the creation of host “self”-peptides, both leading to MHCII peptide loading and expression on macrophage surface [see **Table S1** for a full list of variables and ODE equations from Chang et al. (46)]. This model was simulated over short time scales as the process of antigen presentation, and the *in vitro* studies that were used to develop this model, occurred on a time scale of less than 100 h. This fast time-scale model is linked within macrophages in our longer time-scale model, *GranSim*, that represents approximately 1-year post-infection. This creates a hybrid model that crosses space and time scales ranging from intracellular to tissue and from minutes to months. The inclusion of these intracellular dynamics calibrates the proportion of activated T cells in *GranSim* to match T cell levels that we identified previously in **Figure S1** (21). Below we describe how we linked these two model frameworks.

### Linking Models

There is currently no standard way to link different models, particularly models that are created with different formulations (ODEs, ABMs, PDEs, etc.) (50). We connect the intracellular antigen presentation scale model to the cellular/tissue scale model, *GranSim*, in three ways. **Figure 2** shows how the two models are linked, and **Table 1** shows two linking equations and corresponding parameters for those processes.

1. MHCII transcription depends initially on IFN-γ- derived from CD4+ helper T cells binding to macrophages (50). Levels of binding are thus controlled by the presence of T cells near a macrophage. To link the ODEs and ABM (intracellular to cellular), we determine this concentration by calculating the number of T cells present in the neighborhood of a macrophage at any given time. A *neighborhood* is defined as a Moore neighborhood (nine grid squares) or the two-Moore shell neighborhood (16 grid squares). Macrophages can sample antigen from at least a two-Moore neighborhood (see **Table 1** for values and equations and calculations below).
2. The activation of T cells (CD4+ T helper cells) depends on the presentation of Mtb peptide–MHCII complexes on macrophage surface. The level of complexes seen on macrophage surface depends on the concentration of Mtb antigens present in the surrounding medium (50). Mtb produces a variety of protein and glycolipid antigens. Glycopeptidolipid antigen are some of the most persistent, with only 1%–3% degradation after four days (51). To simulate Mtb antigens, we use dead Mtb as a proxy (dead Mtb are generated directly by macrophages killing them or indirectly by cytotoxic T cells killing infected macrophages). We calculate this concentration directly in *GranSim* by calculating how many dead Mtb are present in the neighborhood of a macrophage (see **Table 1**, and calculations below).
3. To analyze activation levels of T cells, we define how macrophages interact with T cells at given time and space points. A threshold number of Mtb peptide–MHCII on a macrophage surface are needed to activate T cells, and we set a binding threshold of 120 (52, 53). Only macrophages that meet this threshold and are not chronically infected have an ability to activate a T cell (this includes currently infected macrophages and previously infected that cleared their bacterial load). We connect the output of an ODE representing the number of Mtb-MHCII complexes on the surface of a macrophage (**Table S1**, variable $M_{S}^{*}$ ) to each individual macrophage within *GranSim*. For a macrophage to stimulate a T cell, it must be within a one-Moore neighborhood of a macrophage that has a number of Mtb-MHCII complexes that surpasses the binding threshold.

**Table 1:** Equations and parameters for the new linking that are needed to combine the intracellular-scale model and *GranSim*.

Linking Equation description	Equation	
**IFN-γ Receptor Ligand Binding**	
Molar concentration of IFN-γ in a Moore neighborhood of radius 2 around the macrophage (G)	(#. of γ *T cells in* 1 *Moore Neighborhood*)·*l_IFN_*_-γ-_ *_MN_*_1_ + (#. *of* γ *T cells in Moore Shell Radius* 2·*l_IFN_*_-γ-_ *_MN_*_2_)	
**Mtb Antigens**	
Molar concentration of Mtb lipid antigen in a Moore neighborhood of radius 1 around the macrophage (A*)	(#. *of bacteria in* 1 *Moore Neighborhood*)·*l_Mtb-MN_*_1_	
Linking Parameter	Parameter description	Value
*l_IFN_*_-γ-_ *_MN_*_1_	Molar concentration of IFN-γ in a 1 Moore neighborhood around a macrophage	2.0·10^-9^ *M*
*l_IFN_*_-γ-_ *_MN_*_2_	Molar concentration of IFN-γ in a Moore shell of radius 2 around a macrophage	5.7·10^-10^ *M*
*l_Mtb-MN1_*	Molar concentration of Mtb lipid antigen in a 1 Moore neighborhood around a macrophage	4.5·10^-8^ *M*
*l_MHC-bind_*	Number of surface MHCII Mtb-peptide complexes required for binding T cells	1.2×10^2^

See **Figure 2** for a diagram for how this occurs. See calculations below for the values.

### Linking Equation Calculations

First, we calculate the *production of free, extracellular IFN-γ*. We use the following translations to perform the calculation in **Table 1**. IFN-γ produced per T cell: 0.0001 U (54); IFN-γ molecular weight (mature dimer, biologically active): 34 kDa (55); Volume of 1 grid cell: 8.0x10^-12^ L; Number of compartments in 2-Moore shell of radius: 16; IFN-γ U to μg: 2.x10^4^ U = 1 μg (56). Therefore,

1) IFN-γ produced/T cell: 1.5·10^19^ *mol*

2) IFN-γ produced/T cell in one compartment: 1.8·10^-8^ *M*

Thus, the Molar concentration of IFN-γ in a 2-Moore neighborhood (*G*), (where Tg is an IFN-γ -producing T cell), is:

3) *G* = (#. *of Tg*)·2.0·10^-9^ *M* + (#. *of Tg*) 5.7·10^-10^ *M*

Similarly, we calculate *production of extracellular Mtb antigens*: Mtb Antigen mature weight (approx., range 10–40kDa): ~20 kDa (57, 58); Volume of 1 grid cell: 8.0x10^-12^ L; Number of compartments in 1 Moore neighborhood: 9; Mtb biomass: 1.96x10^-13^ g (52, 59); and Fraction of Mtb that are lipids: 0.33 (59).

4) Approx. lipid antigens produced/dead bacteria: 3.2·10^-18^ *mol*

5) Approx. lipid antigens produced/dead bacteria in one compartment: 4.0·10^-7^ *M*

Therefore, the Molar concentration of Mtb lipid antigen in a 1-Moore neighborhood is:

6) *A* ^*^ = (#. *of bacteria*)·4.5·10^-8^ *M*

### Parameter Estimation: Literature and Uncertainty and Sensitivity Analysis

*GranSim* parameter values were estimated from literature [described in detail in (33, 43, 44, 60–62)]. For the intracellular model, ODE parameter and initial condition values were also estimated from the literature [Chang et al. (46). and shown again in **Tables S2** and **S3**]. Linking parameters are calculated as shown above. If data were not available, we implemented uncertainty analysis using a Latin hypercube sampling scheme (LHS) [reviewed in (63, 64)]. We use LHS to sample and find parameters for the ODEs that represent the dynamics within the macrophages (intracellular) and also to calibrate *GranSim* to experimental datasets. Extensive data on numbers of macrophages, Mtb, and T cells were provided by the Flynn lab as previously described (21, 33, 43, 49, 65). To narrow down parameter ranges and mechanisms of interest, we identify critical parameters that map to specific model mechanisms that impact model outputs. To do this, we take a two-step process: we pair LHS with Partial rank correlations (PRC) analysis (sensitivity), which allows us to quantify the correlation of model outputs with parameters, including those with non-linear relationships (63). We do this by calculating partial rank correlation coefficients (PRCCs) that are between -1 and 1 and indicate the strength of the correlations. These are nonlinear correlations, so that is why they are ranked. PRCCs p-values were corrected for multiple testing using Bonferroni (66).

### Mtb-Mediated Inhibition of Antigen Presentation

Mtb may inhibit MHCII Mtb-antigen presentation within macrophages by interfering with MHCII mRNA transcription, antigen processing, MHCII maturation, and/or MHCII peptide loading (30, 31, 46). To determine if these fast-time and short physiological scale events manifested at the granuloma scale, we tested the effects of inhibiting these four processes on MHCII antigen-presentation on downstream T cell activation. To do this, we examined a range of rates of down regulation of antigen processing (**Table S4**) to be used with Michaelis–Menten dynamics (**Table S5**), as done previously in (46).

### T Cell Spatial Characteristics

**Figure 3:**
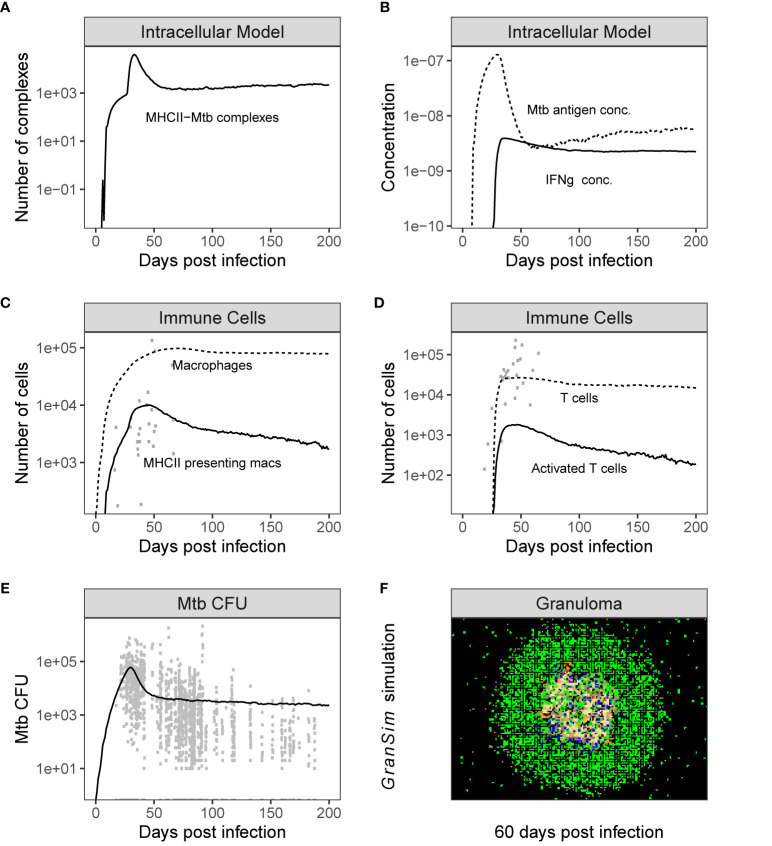
Simulation output is consistent with NHP data. Time series plots showing the dynamics of both individual model outputs from the combined Multi-scale model. Panels **(A, B)** shows the variables in the intracellular model over time, and Panel **(C, D)** show the time series of total populations of immune cells [shown together with non-human primate (NHP) datasets from Wessler et al. (67)]. Panel **(E)** shows the CFU [shown together with NHP data sets from Wessler et al. (48)] and Panel **(F)** is a time series snapshot at day 60 of the Multi-scale *GranSim* granuloma model, 2x2 mm scale. Cell types in Panel **(F)**: Macrophages: resting (green), active (blue), infected (orange), chronically infected (red); T cells: Mtb-specific (dark pink), non Mtb-specific (light pink); caseation (tan); extracellular bacteria (dark yellow).

Based on previous work, we have identified that the spatial organization of granulomas can be a determinant in granuloma outcomes (34). Thus, we separately explored the spatial organization of granulomas and the role that may play in reducing the number of Mtb-responsive T cells. To do this, we use the combined ODE-ABM multi-scale model with no inhibition of antigen-presentation processes. From these 500 simulations, 37 scenarios were removed as the bacterial infection did not occur or did not generate T cells within the first 50 days, leaving 463 simulated granulomas to be analyzed. (Results using similar runs gave similar results.) We chose a replicate run of *GranSim* that fit the median characteristics of the 463 *GranSim* simulations over the time scale of 200 days post infection (**Figure S2**). These characteristics include: numbers of macrophages, Mtb counts, proportion of activated T cells, and average presentation of antigenic peptide–MHCII complexes on macrophage surface (see example median runs chosen based on these characteristics in **Figure 3**). We calculated minimum, average, and maximum distance of immune cell populations based on their relation to the granuloma center of mass [as in Renardy et al. (68)].

### Modeling Recruitment of Mtb-Specific Versus Non-Specific T Cells

All T cells in the model (three functional classes: IFN-γ -producing T cells (Tgammas), cytotoxic T cells (Tcyts), and regulatory T cells (Tregs)) are recruited into *GranSim* as either specific or non-specific T cells (62, 69). At each time step, at each vascular source from where cells are recruited, T cell classes are recruited as determined by the chemokine concentrations at each vascular source. Three parameters (Tgam.probCognate, TCyt.probCognate and Treg.probCognate) determine the ratio of Mtb-specific to non-specific T cells. Patankar et al. showed in mice granulomas that 5%–20% of T cells are Mtb-specific (70). Here, we varied the frequency of each Mtb-specific T cell class from 1%-25% to capture a potential larger range occurring within primates. Both specific and non-specific T cells enter the grid in a T_h_0 state that requires further stimulation to fully differentiate and perform effector function. Non-specific T cells remain in a T_h_0 state in the granuloma throughout the simulation and do not have the ability to kill Mtb. Both Mtb-specific and non-specific T cells have the ability to move on the grid and die from old age, or *via* TNF induced apoptosis. Macrophages with sufficiently bound surface MHCII receptors (larger than the MHCII binding threshold) can activate Mtb-specific T cells in their neighborhood.

### Computer Simulations and Visualization

The 14 equation ODE model describing intracellular antigen presentation dynamics is solved within each macrophage within *GranSim* along with the new equation terms linking models for each model time step. If the number of surface bound MHC II receptors of a macrophage is at or above a threshold (parameter MHCII Binding Threshold), then any specific T_h_0 cell in the 1-neighborhood of a macrophage’s transitions from the T_h_0 state to an active state. *GranSim* was implemented in C++ with Boost and FFTw libraries. Partial differential equations describing diffusion are solved using Alternating Direction Explicit method. MHCII dynamic ODEs are solved using Runga-Kutta 4 method. Simulations for parameter sweeps were run without graphical visualization. The graphics visualization version was then used to load saved simulation states and generate graphics images to visually track granuloma formation. Computational model simulations were performed on XSEDE’s Comet cluster and NERSC’s Cori and Edison systems. For details on the system we use see https://www.sdsc.edu/support/user_guides/comet.html.

## Results

Our goal is to study three key hypotheses explaining the relatively low frequency of T cells producing cytokines in TB granulomas. We test each hypothesis individually using both temporal and spatial modeling and appropriate control studies. In some instances, we have data that have been derived herein to provide support for our predictions.

### Hypothesis 1: T Cells Are Down-Regulated Directly by the Action of Mtb

To explore this hypothesis, we include the role of intracellular dynamics of MHCII-mediated antigen presentation within macrophages within our multi-scale model of granuloma formation, *GranSim*. We then test four different types of down-regulation by Mtb on antigen presentation individually and combine to observe the effects on downstream T cell activation. As a control, we implement no downregulation (as in the *GranSim* model without the submodel in place).

**Figure 4:**
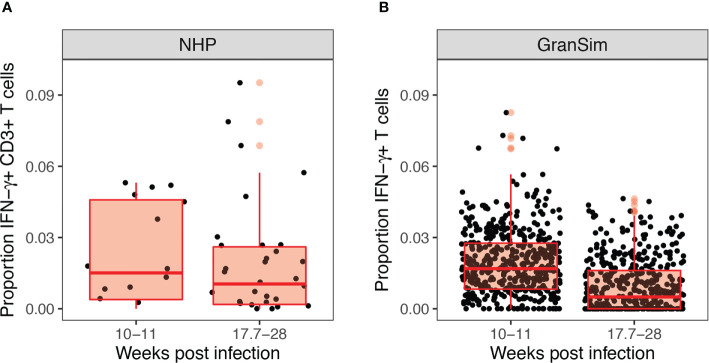
Experimental and computational models both reveal low levels of Mtb-responsive cells producing interferon-gamma (IFN-γ) within the granuloma. Panel **(A)** shows the experimental proportion of T cells exhibiting an IFN-γ response in non-human primates from Gideon et al. (21), from 50 granulomas derived from 12 non-human primates (NHPs). Panel **(B)** shows our simulated prediction of the proportion of IFN-γ producing T cells in 500 simulated granulomas over the 28 weeks course of infection grouped to match the NHP dataset.

We performed 500 simulations using a wide range of biologically relevant parameters values (generated by LHS, see Methods). **Figure 3** shows outputs for both the intracellular scale model and cellular/tissue scale model for different variables of interest. Model dynamics agree with datasets derived from NHP studies on granulomas and other *in vitro* studies for intracellular dynamics (71–73). We also compared our model predictions to *GranSim* without MHCII presentation dynamics to confirm our model behaved accurately (positive control) (with values in **Table S5** set to 1, **Figure S3**). Lastly, we calculated the numbers of Mtb-responsive T cells for 500 granulomas and compared it with data derived from 50 granulomas from an NHP study (**Figure 4**) (21).

#### MHC Self-Derived Peptides Increase T Cell Activation When MHCII Peptide Loading Is Inhibited

We performed a sensitivity analysis on the intracellular-scale model (without Mtb assisted downregulation of MHCII presentation) and identified several parameters correlated with MHCII-mediated antigen presentation (**Figure S4**). We found that increasing Mtb antigen processing rates by macrophages leads to increased levels of MHCII-Mtb presentation, but that these effects waned by 75 days post infection (**Figure S4**). This matches general trends predicted in (46). We also found that increases in Mtb antigen degradation or increases in MHCII-Mtb antigen dissociation leads to decreased MHCII-Mtb presentation on the surface of macrophages, also waning by 75 days post infection (**Figure S4**). At the 75 days post in infection, Mtb levels within granulomas have leveled off, likely leading to diminishing returns for macrophage MHCII-Mtb presentation (**Figure 3A**). However, we did not identify correlations of antigen presentation parameters with a critical downstream effect, namely, levels of activated T cells within simulated granulomas. Previously a number of labs had identified different pathways in the antigen processing and presentation of MHCII-peptides by macrophages that were inhibited by Mtb (30, 31). Chang used the single cell model to study how Mtb affects certain processes of MHCII presentation and specifically studied Mtb interference of MHCII transcription, MHCII maturation (CIITA translation rate), antigen processing, and MHCII peptide loading (46). We explored these same four processes within the linked multi-scale model, where we tested a range for the maximum rate of Mtb down-regulation of each of the processes, at saturating bacterial levels, ranging from 25% to 100%.

Of the four processes that we studied for inhibition by Mtb, we found that reducing MHCII peptide loading was the only process that had a significant effect on T-cell activation levels (**Figure S5**). When Mtb acts to down regulate MHC II peptide loading, as the degradation rate of peptide-MHC complexes increases, all T cell classes showed increased levels of activation, but only after 100 days post infection (not shown, p < 0.0005). At this point in the infection as Mtb levels plateau (see **Figure 3E**, day 75), degrading peptide-MHC complexes may help remove MHCII complexes loaded with host “self”-peptide. Continually degrading these complexes may help cycle through peptides quicker, making it more likely that MHCII are loaded with Mtb-peptides. This cycling through of peptides may be the only way to maintain a certain threshold of MHCII complexes presenting Mtb when peptide loading is greatly inhibited and Mtb levels have fallen. However, this contribution is small when observed as a proportion of activated T cells (**Figure S5**). As a control, we also performed a sensitivity analysis on Mtb-induced downregulation of MHCII presentation for each of the processes, varying down regulation from 1% to 100% (**Figure S6**). Any significant correlations with T-cell activation were small and transient, suggesting that these processes contribute very little.

Although previous wetlab and modeling studies showed that mycobacteria significantly inhibit antigen presentation processes, the focus of these *in vitro* studies was on less than 100 h (30, 31, 46). However, our results suggest that bacterial mechanisms alone do not account for the observed low T-cell responsiveness levels of cytokine production observed in NHPs at a granuloma scale (21).

### Hypothesis 2: Spatial Organization of Granulomas Affects the Ability of T Cells to Reach Macrophages and Thus Be Activated *Via* Antigen Presentation

We test a second hypothesis, namely that the spatial arrangement of cells within granulomas may create insufficient numbers of interaction opportunities between macrophages and T cells. This would imply that even if Mtb downregulation of processes is important, the chances for impact are few. In other words, granuloma spatial characteristics may contribute to low T cell responsiveness (26).

To explore this idea, we used a two-pronged approach analyzing both experimental and simulated granulomas to better understand the spatial arrangements of immune cells. First, we randomly selected four experimental immunohistochemistry (IHC) images derived from four distinct NHP granulomas to directly identify and quantify the spatial organization between T cells and macrophages. We applied a novel approach using Geographical Information Systems Technology (GIS) similar to what we have done previously to analyze cell composition of granulomas [see Methods and Pienaar et al. (74)]. Here we not only identify the T cells and macrophage populations, but we additionally quantified the interaction overlap between T cells and macrophages, defined where these two cell boundaries intersect on the IHC image. We found that T cell-macrophage interactions occurred for, on average, only about 9.75% of the T cells identified (median: 8.6%, StDev: 4.5%), for at least the four granuloma that we examined (See **Table 2**).

**Table 2:** Geographical Information Systems Technology (GIS) analysis identifies numbers of cells of two types, and numbers of contacts from four immunohistochemistry (IHC) granulomas.

Granuloma	CD3	CD11c	CD3/CD11c	Ratio Contacts to T cells
9714_30	4,969	9,876	491	0.098
17613_37	15,448	3,943	1,127	0.073
17613_51	8,612	4,496	1,397	0.162
JF13-18	4,800	2,975	48	0.010

For the four IHC images of granulomas analyzed in **Figure 1**, we used GIS to count the T Cells (CD3) and Macrophage (CD11c), cell distributions and their ratios. We also identified where the two cell types intersected. Intersections are defined as cell boundaries that touched or overlapped on the IHC image.

**Figure 5:**
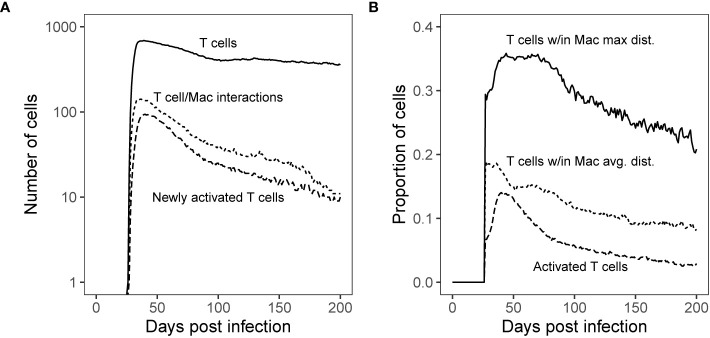
Simulated T cell/Macrophage Interactions in time Shows Spatial Analysis of Simulated Granulomas. Panel **(A)**: Median count T cells over the course of Mtb infection compared with the median count of distinct T cells that interact with macrophages and new T cell activations per time step. Panel **(B)**: Proportion of T cells found within the maximum boundary and average distance of antigen-presenting macrophages (measured from the granuloma center of mass) as compared with the proportion of total activated T cells (all activated T cells are Mtb-specific). These distances are shown spatially in **Figure 6**.

Next, we used simulated granulomas and performed the same analysis to predict spatial locations of interactions between T cells and activated macrophages. For this, we simulated *GranSim* and controlled for bacterial inhibition as a factor by removing all possible Mtb-mediated down-regulation of MHCII presentation processes. We performed 500 simulations of granulomas and ran them out for 200 days post virtual infection. For each day during the virtual infection, we calculated median counts of T cells, new T-cell activation events, and numbers of distinct T cells that interacted with at least one macrophage in each of the 1-day intervals. Similar to the NHP granuloma T cell- macrophage interactions (**Figure 1**), we observed a similar order of magnitude difference between numbers of T cells and T cell-macrophage interactions, as well as new T cell activation events (**Figure 5**). This last feature is something we can uniquely track in *GranSim*. Distinct T cells that interacted with at least one macrophage occurred for only about 10% (with a range of 0%–22%, StDev: 4.4%) of all T cells identified for a given time point at 11-weeks post virtual infection and slowly declined to about 5% at 25-weeks post virtual infection (**Figure S7**). As a control, we compared model predictions of T cells activation to *GranSim* with varying ‘flexibility of T-cell density’ as follows. We allowed the maximum number of T cells that can fit within one grid compartment to vary from 2 to 5, 2 being the default negative control (**Figure S8**). Increasing the maximal allowable T cell density within a grid space did increase the proportion of activated T cells, but resulted in values that did not capture most of the data observed in the NHP study (21). We also performed a sensitivity analysis on T cell density over the same range (**Figure S9**). Increasing the density of T cells is correlated with increasing T cell activation. However, increasing T cell density did not result in higher Mtb clearance. As T cell density increased, we saw a stabilizing effect on Mtb CFU after day 50 (**Figure S10**). This is likely due to increased crowding on the grid, where T cells can slip by, but larger macrophages become stuck, making it more difficult to find and kill Mtb.

#### The Majority of T Cells Are Not Being Stimulated

Secondly, we test that the majority of T cells are not being stimulated. Both wetlab and computational studies to support the idea that within granulomas, T cells are often not sufficiently close to macrophages to become activated (32, 33). We can use our simulations to determine how far each cell type is from the center of a granuloma. For each day of the virtual infection, we used *GranSim* to calculate median proportions of T cells found within both the average and maximum distance of macrophages from the granuloma center of mass [as in Renardy et al. (68)]. These numbers are compared with the proportion of activated T cells (**Figure 5B**). Within our simulations, roughly a third of T cells travel deep enough within the granuloma to have the possibility of reaching activated macrophages. Of these, about half made it past the average distance of T cell stimulating macrophages from the granuloma center of mass, increasing their chance of encountering a T cell stimulating macrophage (**Figure 5B**).

**Figure 6:**
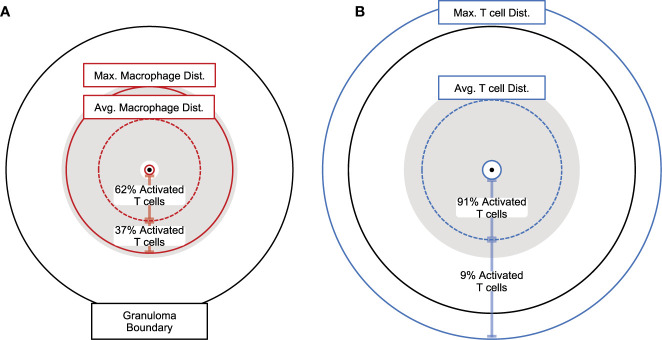
Spatial analysis of granulomas showing distances between macrophages and T cells. Proportional distances of average maximum, and minimum **(A)** activated macrophages presenting antigen or **(B)** Mtb-specific T cells from the granuloma center of mass (7-weeks post infection). Maximum and minimum distances shown as solid circles, average distances show as dotted circles. Gray shaded areas encompass all activated Mtb-specific T cells present. Percent of all activated T cells is shown between minimum and average, as well as average and maximum distances for both **(A)** activated macrophages presenting antigen and **(B)** Mtb-specific T cells.

To get a more detailed look at the simulated granulomas and spatial distributions of cells, we extracted the coordinates of all macrophage and T cell agents in our simulated granuloma (see Methods). In **Figure 6**, the distribution of macrophages (**Figure 6A**) and T cells (**Figure 6B**) are drawn in relation to the granuloma center of mass. The area shaded gray is the distribution of activated T cells. At the height of T cell activation (occurring about 7-weeks post infection), almost all activated T cells (**Figure 6A**, shaded gray) can be found within the spatial region of macrophages that are able to stimulate T cells. That is, very few activated T cells are found near activated macrophages. This distribution of activated T cells can be divided further, with two thirds residing within the average distance of activated macrophages from the granuloma center of mass. About 90% of the activated T cells are found within the average Mtb-specific T cell distance from the granuloma center of mass (**Figure 6B**). In general, the distribution of activated T cells closely follows the distribution of activating macrophages. Low T cell stimulation, taken together with limited T cell access to macrophages and an observed increase in T cell activation by increasing T cell density suggest that spatial mechanisms play a major role in the observed low T-cell responsiveness levels of cytokine production observed in NHPs at a granuloma scale.

### Hypothesis 3: The Majority of T Cells Within Granulomas Are Non-Mtb Specific

**Figure 7:**
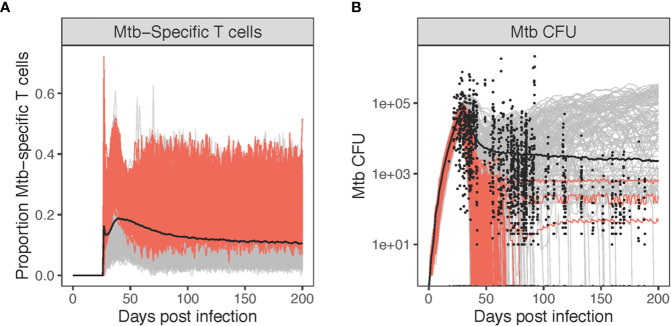
Varying levels of Mtb-specificity in granuloma T cells to match non-human primate (NHP) studies. Panel **(A)**: The proportion of Mtb-specific T cells over the course of virtual infection. Panel **(B)** shows the CFU [shown together with 1,994 NHP granulomas (black circles)]. Highlighted in red are simulations where the probability of Mtb-specific T-cell classes recruited are allowed to exceed 10% (up to 25%). All other simulations are gray (1%–10%).

To test the hypothesis that the majority of T cells within granulomas are non-Mtb Specific, we focus on the spatial distribution of Mtb-specific T cells (**Figure 6**). Given previous studies showing that T cells are recruited to macrophages indiscriminately (36–38), we expanded upon our study of the composition of Mtb-specific T cells within simulated granulomas. We use our multi-scale model to determine the proportion of Mtb-specific T cells within granulomas over the course of infection as compared to non-specific T cells present by comparing which frequencies match the dataset derived from NHP. To do this, we varied the frequency of Mtb-specific T cell classes from 1%-25% to capture a potential larger range occurring within primates (70). For each day of virtual infection, we used *GranSim* to calculate median, numbers, and proportions of Mtb-specific T cells versus non-specific T cells found within simulated granulomas (**Figure 7**). Within our simulations, non-Mtb-specific T cells greatly outnumber Mtb-specific T cells (**Figure 7A**). The proportion of Mtb-specific T cells peaks at around day 40 at about 20% (**Figure 7A**) and declines to about 10% by 200 days post virtual infection.

Given the low number of Mtb-specific T cells within granulomas, we looked at how these simulations matched with NHP Mtb CFU data (**Figure 7B**). Highlighted in red are simulations where the probability of Mtb-specific T-cell classes recruited is allowed to exceed 10% (all other simulations are gray). Allowing recruitment of a great portion of Mtb-specific T cells results in the majority of granulomas sterilizing by day 100 (226/229 = 0.987). The red simulations (**Figure 7B**) also overlap with relatively few of the NHP CFU data points. This suggests that low levels of Mtb-specific T cells are what likely is present within granuloma, leading to the observed NHP Mtb CFU datasets (**Figure 7B**). Any further increases in levels of Mtb-specific T cells within granulomas leads to sterilization of 99% of all granulomas, which is not a typical outcome; more typically around 50% (42). As a control, we also performed a sensitivity analysis on Mtb-specific T cell proportions for each class of T cells represented in *GranSim*, varying specificity from 1% to 25% (**Figure S9**). The probability of IFN-γ producing T cells being Mtb-specific had the greatest effect on T cell activation and Mtb clearance. When T cells were first recruited, they had a strong, positive effect on the number and proportion of activated T cells. By day 50, this shifted to a negative correlation, due to the small numbers of activated T cells seen at this stage of infection. Increasing Mtb-specific IFN-γ producing T cells also increased the clearance of Mtb, matching the sterilization observed in **Figure 7B**.

### Combining the 3 Hypotheses

We performed a sensitivity analysis, varying parameters for all three hypotheses at the same time to search for combined effects, using the previously specified parameter ranges (**Figure S11**). In general, correlation patterns for these input parameters in combination were similar to those seen when simulated separately (**Figures S6** and **S9**). One difference we observed is that parameters controlling the proportion of Mtb-specific T cells continued to have a positive effect on the number and proportion of activated T cell past day 50. Together, these results suggest that the proportion of Mtb-specific T cells influences the observed low T-cell responsiveness levels of cytokine production observed in NHPs at a granuloma scale and the other hypotheses may help extend these effects over time.

## Discussion

Over 90% of Mtb infections in humans are well controlled and asymptomatic, known as Latent TB infection (LTBI), indicating that the immune response to Mtb, which is characterized by granuloma formation, is relatively successful at containing infection. Cynomolgus macaques also present with active or latent TB. As the majority of granulomas in both latent and even active NHPs eventually sterilize (42), this means that granulomas (on an individual basis) have the ability to clear infection. It is surprising that low T-cell activation levels through measuring IFN-γ and other cytokine responses have been observed within non-human primate granulomas (21). There are a number of hypotheses as to why low numbers of responsive T cells might be present in TB granulomas, including T cell exhaustion, Mtb-mediated downregulation of antigen presentation by macrophages, the spatial organization of cells within granulomas and the presence of non-Mtb specific T cells. We previously explored exhaustion; however, our results indicate that it cannot explain the observed low levels (26). Herein we explored the other three hypotheses as to why the numbers of Mtb-specific T cells are low. First, we focused on macrophages and their role in this outcome: We asked whether Mtb was down-regulating MHCII presentation of Mtb antigens and whether that reduced T cell activation. At the scale of the entire granuloma, we did not see significant differences in MHCII presentation of Mtb antigens by macrophages, with or without Mtb down-regulating of MHCII presentation (**Figure S5**). Previous work suggesting that inhibition by Mtb was a key player in reducing MHCII presentation were based on studies that spanned time scales of 1-100 h, while granulomas survive for months to years (30, 31, 46). The dynamics observed on the scale of a few hours may be washed out given the extended lifespan of macrophage and Mtb- dynamics with granulomas. Further, even if large scale reductions in antigen presentation are occurring, our further studies indicate that there are insufficient interactions occurring between T cells and macrophages for that to manifest as a key factor.

Although we did not see a significant decline in MHCII presentation of Mtb antigens on the surface of macrophages at any given time, we did observe that only 5-10% of macrophages in our simulations were capable of activating T cells. In *GranSim*, only macrophages that have contact with both IFN-γ (*via* Stat 1) and Mtb or TNF (*via* NFkB) can present Mtb antigens *via* MHCII. Macrophages must continually receive those stimuli until they surpass an MHCII surface level threshold required to activate T cells (53). These activated macrophages were spatially located mostly within the center of our simulated granulomas, where they would have access to Mtb and Mtb antigens. Since many macrophages did not receive stimuli necessary for MHCII Mtb-antigen presentation, it is not surprising that the inhibition of antigen presentation by Mtb was minimal at the tissue scale. It is also possible that since our model does not account for the effect of chemokines attracting T cells to antigen presenting cells, and Mtb antigens do not include secreted antigens, antigen concentration and T cell numbers responsive to infection may be underestimated. However, in previous work, we have examined this idea of APCs secreting chemokines to attract T cells, and have shown that it leads to tremendous crowding around APCs, limiting stimulation (75).

Since direct inhibition of antigen-presentation by Mtb was insufficient to reduce T cell responsiveness, we explore the second hypothesis of how granuloma spatial structure may affect T cell activation. Our analysis of four HIC images from NHP granulomas suggest that there are limited interactions between T cells and CD11c+ macrophages within granulomas, and further analysis with additional granulomas is warranted. Previous studies have also shown that within granulomas, T cells are highly motile but restricted by space, with movement occurring mostly at the borders of the granuloma (32, 33, 37). In addition, the typical structure for a granuloma is a lymphocytic cuff surrounding macrophages and other cells including bacteria and more centralized necrosis (76, 77). All activated macrophages within our simulations are located near the center of granulomas and most T cells are unable to reach them. In fact, only about 5% of all T cells in *GranSim* interact with macrophages at any given time. Activated T-cell life spans are short (on average 3 days), so large numbers are unlikely sustainable. It should be noted that T cells can have functions other than cytokine production, and our study used only data on T cell production of IFN-γ. Assessing other T cell effector functions, such as other cytokines and cytotoxic potential, could result in an increase in numbers of T cells that are responsive to the infection. Within this study, low T cell stimulation, taken together with limited T cell access to macrophages and an observed increase in T cell activation by increasing T cell density suggest that the structural organization of the granuloma seems to impact the T cells in a significant manner. However, that does not rule out that other factors are playing a simultaneous role. What we have shown is that the spatial effects are necessary condition for this reduction in T cell responsiveness; however, it could certainly hold true that these other factors are also playing a role in augmenting those dynamics, albeit less significant. Our combined analysis of all three hypotheses simultaneously confirmed this.

Continuing with the idea of limited T cell-macrophage interaction dynamics, we explore the issue of recruitment of Mtb-specific T cells into granulomas. Previous studies have shown that T cells localize to and are rapidly recruited into mycobacterial granulomas in the absence of antigen recognition (36–38). One study found around 5-20% of CD4+ T cells recognize Mtb-infected macrophages by 19- to 22-weeks post infection (70). Comparatively in *GranSim*, 10%–20% T cells are Mtb-specific T cells 7- to 28-weeks post infection, with only Mtb-specific T cells given activation capabilities. When these percentages increase in *GranSim*, T cell activation goes up and the vast majority of granulomas are sterilized. Given that such a small percentage of T cells can recognize Mtb antigen, along with the limited migration of T cells, these two factors combine to make T-cell activation a rarer occurrence than one would expect. As most granulomas can sterilize or greatly reduce bacterial numbers, this level of T cell activation may generally be effective in conjunction with other help from the immune response. However, if specificity and location could be affected in a direct way, the numbers of activated T cells would increase and infection would likely be cleared within all granulomas. An appropriate vaccine could lead to this outcome.

## Data Availability Statement

The raw data supporting the conclusions of this article will be made available by the authors, without undue reservation.

## Ethics Statement

The animal study was reviewed and approved by University of Pittsburgh Institutional Animal Care and Use Committee (IACUC).

## Author Contributions

DK, JF, and JL designed the study. NLG, JTM, SE, and JF carried out the experiments. JAM and JB analyzed the data. JAM and DK drafted the manuscript. All authors contributed to the article and approved the submitted version.

## Funding

This research was supported by R01AI123093 awarded to DK and JF and R01HL110811 awarded to DK, JL, and JF. JAM is supported by the National Science Foundation Graduate Research Fellowship Program under Grant No. DGE-1256260. Any simulations also use resources of the National Energy Research Scientific Computing Center, which is supported by the Office of Science of the U.S. Department of Energy under Contract No. ACI-1053575, the Extreme Science and Engineering Discovery Environment (XSEDE), which is supported by National Science Foundation grant ACI-1548562, and National Energy Research Scientific Computing Center (NERSC), a U.S. Department of Energy Office of Science User Facility operated under Contract No. DE-AC02-05CH11231.

## Conflict of Interest

The authors declare that the research was conducted in the absence of any commercial or financial relationships that could be construed as a potential conflict of interest.
