# Corrigendum: Spatial Organization and Recruitment of Non-Specific T Cells May Limit T Cell-Macrophage Interactions Within *Mycobacterium tuberculosis* Granulomas

**DOI:** 10.3389/fimmu.2021.790557

**Published:** 2021-11-22

**Authors:** Jess A. Millar, J. Russell Butler, Stephanie Evans, Nicole L. Grant, Joshua T. Mattila, Jennifer J. Linderman, JoAnne L. Flynn, Denise E. Kirschner

**Affiliations:** ^1^ Department of Epidemiology, University of Michigan, Ann Arbor, MI, United States; ^2^ Department of Computational Medicine and Bioinformatics, University of Michigan, Ann Arbor, MI, United States; ^3^ Department of Health and Biomedical Sciences, AdventHealth University, Orlando, FL, United States; ^4^ Department of Microbiology and Immunology, University of Michigan Medical School, Ann Arbor, MI, United States; ^5^ Department of Infectious Diseases and Microbiology, University of Pittsburgh, Pittsburgh, PA, United States; ^6^ Department of Chemical Engineering, University of Michigan, Ann Arbor, MI, United States; ^7^ Department of Microbiology and Molecular Genetics and the Center for Vaccine Research, University of Pittsburgh, Pittsburgh, PA, United States

**Keywords:** T cell, macrophage, *Mycobacterium tuberculosis*, lung, computational model, granuloma

In the original article, there was a mistake in **Figure 1** as published. The original image included cell types not referenced in the paper and data from an analysis was not included in the paper. The corrected **Figure 1** legend appears below.

“Immunohistochemistry analysis of four non-human primates (NHP) granulomas [shown in Panels **(A–D)**] examining spatial distributions of both T cells and macrophages, and also where they intersect. Four distinct, randomly chosen granuloma images with extracted cell distributions. Column 1 shows the immunohistochemically stained preparation for CD3 (green), CD11c+ macrophages (red), and nuclei (dark blue). White points represent Geographical Information Systems Technology (GIS) analyses of these images revealing cell locations for T cells (Column 2), macrophages (Column 3), and their intersections (Column 4), as follows. Rows represent four distinct granulomas. The data for the cell numbers in these granulomas are given in **Table 2**. On average, about 9.75% (median 8.6%, StDev is 4.5%) of T cells interacted with macrophages.”

In the original article, there was a mistake in **Table 2** as published. The original image included data from an analysis of cell types not described in this publication and non-standard non-human primate identification numbers. The corrected **Table 2** appears below.

For the four IHC images of granulomas analyzed in **Figure 1**, we used GIS to count the T Cells (CD3) and macrophages (CD11c), cell distributions and their ratios. We also identified where the two cell types intersected. Intersections are defined as cell boundaries that touched or overlapped on the IHC image.

Nicole L. Grant was not included as an author in the published article. The corrected **Author Contributions Statement** appears below.

“DK, JF, and JL designed the study. NLG, JTM, SE, and JF carried out the experiments. JAM and JB analyzed the data. JAM and DK drafted the manuscript. All authors contributed to the article and approved the submitted version.”

In the original article, there was an error. In the **Methods** section, the number of macaques that the granulomas in **Figure 1** were obtained from and their weeks post-infection were incorrectly included, antibodies for a cell type not analyzed in this study were included, and a microscope that used for imaging was excluded. A correction has been made to **Methods**, **Immunohistochemistry and Imaging**, paragraph 1:

“Four randomly selected, formalin fixed paraffin embedded (FFPE) granulomas were derived from 3 cynomolgus macaques (*Macaca fascicularis*), necropsied at approx. 10-11 weeks post infection (**Figures 1A–D**), and were deparaffinized and antigen retrieval was performed as previously indicated (35). Granulomas were stained with cocktails of antibodies including polyclonal rabbit anti-CD3 (Agilent Technologies, Santa Clara, CA), IgG2a mouse anti-CD11c (clone 5D11; Leica Microsystems, Buffalo Grove, IL). Primary antibodies were labeled with fluorochrome-labeled secondaries including anti-isotype (IgG2a) specific antibodies (Jackson ImmunoResearch, West Grove, PA). Coverslips were mounted with Prolong Gold with DAPI (ThermoFisher Scientific) and the sections were imaged on an Olympus FluoView confocal microscope (Center Valley, PA) or Nikon e1000 epifluorescence microscope (Nikon Instruments, Melville, NY) with Nikon NIS Elements (Nikon Instruments).”

**Figure 1:**
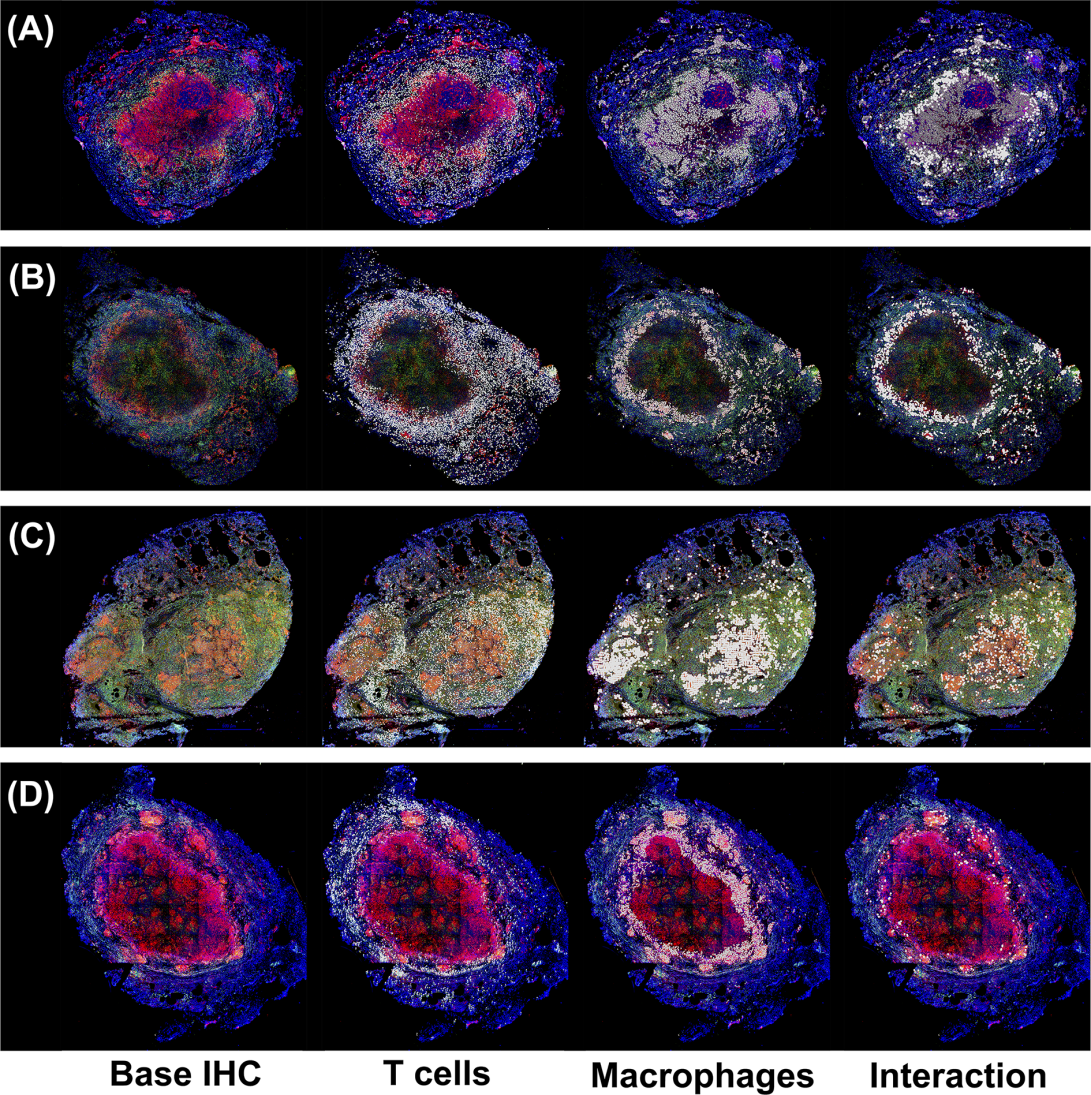
Immunohistochemistry analysis of four non-human primates (NHP) granulomas [shown in Panels **(A–D)**] examining spatial distributions of both T cells and macrophages, and also where they intersect. Four distinct, randomly chosen granuloma images with extracted cell distributions. Column 1 shows the immunohistochemically stained preparation for CD3 (green), CD11c+ macrophages (red), and nuclei (dark blue). White points represent Geographical Information Systems Technology (GIS) analyses of these images revealing cell locations for T cells (Column 2), macrophages (Column 3), and their intersections (Column 4), as follows. Rows represent four distinct granulomas. The data for the cell numbers in these granulomas are given in Table 2. On average, about 9.75% (median 8.6%, StDev is 4.5%) of T cells interacted with macrophages.

In the **Results** section, we included a statistic for a cell type not analyzed in this study. A correction has been made to **Results, Hypothesis 2: Spatial Organization of Granulomas Affects the Ability of T Cells to Reach Macrophages and Thus Be Activated Via Antigen Presentation**, paragraph 2:

“We found that T cell-macrophage interactions occurred for, on average, only about 9.75% of the T cells identified (median: 8.6%, StDev: 4.5%), for at least the four granuloma that we examined (See **Table 2**).”

**Table 2:** Geographical Information Systems Technology (GIS) analysis identifies numbers of cells of two types, and numbers of contacts from four immunohistochemistry (IHC) granulomas.

Granuloma	CD3	CD11c	CD3/CD11c	Ratio contacts to T cells
9714_30	4,969	9,876	491	0.098
17613_37	15,448	3,943	1,127	0.073
17613_51	8,612	4,496	1,397	0.162
20612_29	3,284	5,535	197	0.060

The authors apologize for these errors and state that they do not change the scientific conclusions of the article in any way. The original article has been updated.

## Publisher’s Note

All claims expressed in this article are solely those of the authors and do not necessarily represent those of their affiliated organizations, or those of the publisher, the editors and the reviewers. Any product that may be evaluated in this article, or claim that may be made by its manufacturer, is not guaranteed or endorsed by the publisher.

