# Supplementary material for: Spatial Organization and Recruitment of Non-Specific T Cells May Limit T Cell-Macrophage Interactions Within Mycobacterium tuberculosis Granulomas
Source: Front Immunol. 2021 Jan 20;11:613638. doi: 10.3389/fimmu.2020.613638 (PMC7855029; doi:10.3389/fimmu.2020.613638)
Supplement: Supplementary file 1 [file DataSheet_1.pdf]

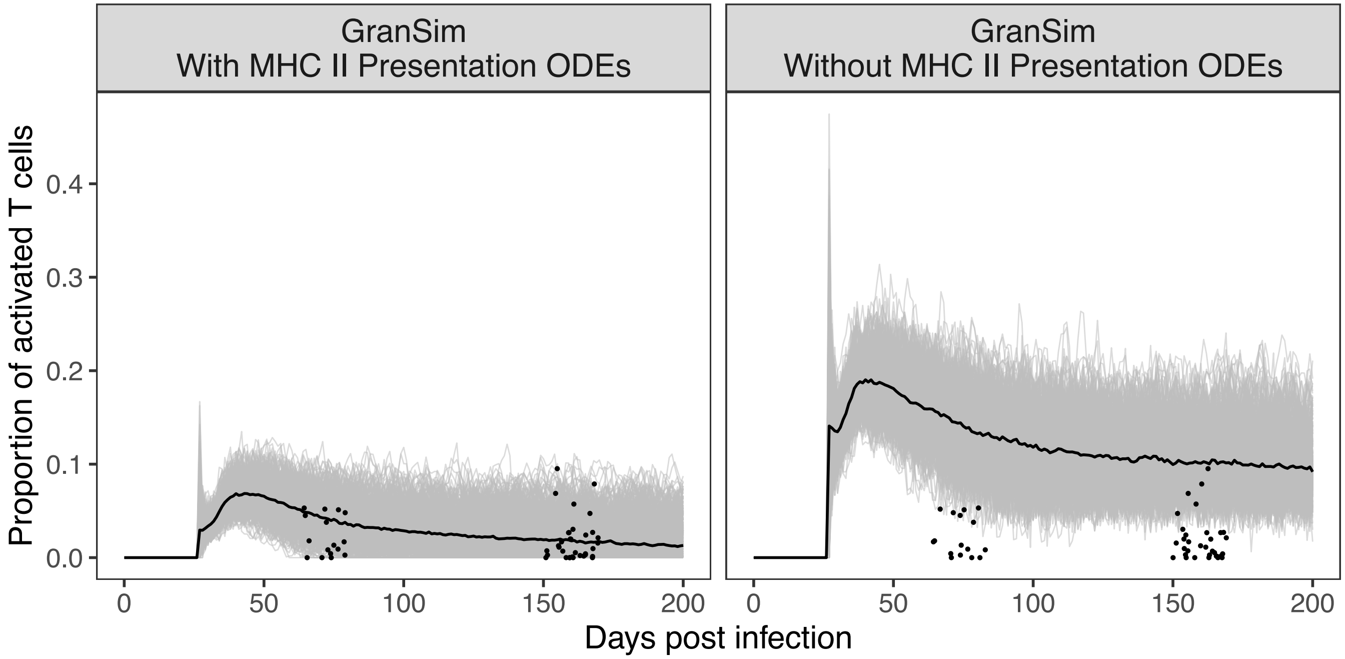

Figure S1: Comparison of proportion of activated T cells in *GranSim* simulations with MHCII presentation ODEs and without. Median is shown in black, with simulation runs shown in gray. NHP data is overlaid and jittered over ten-day ranges (as only ranges were available) for each output (Gideon et al., 2015).

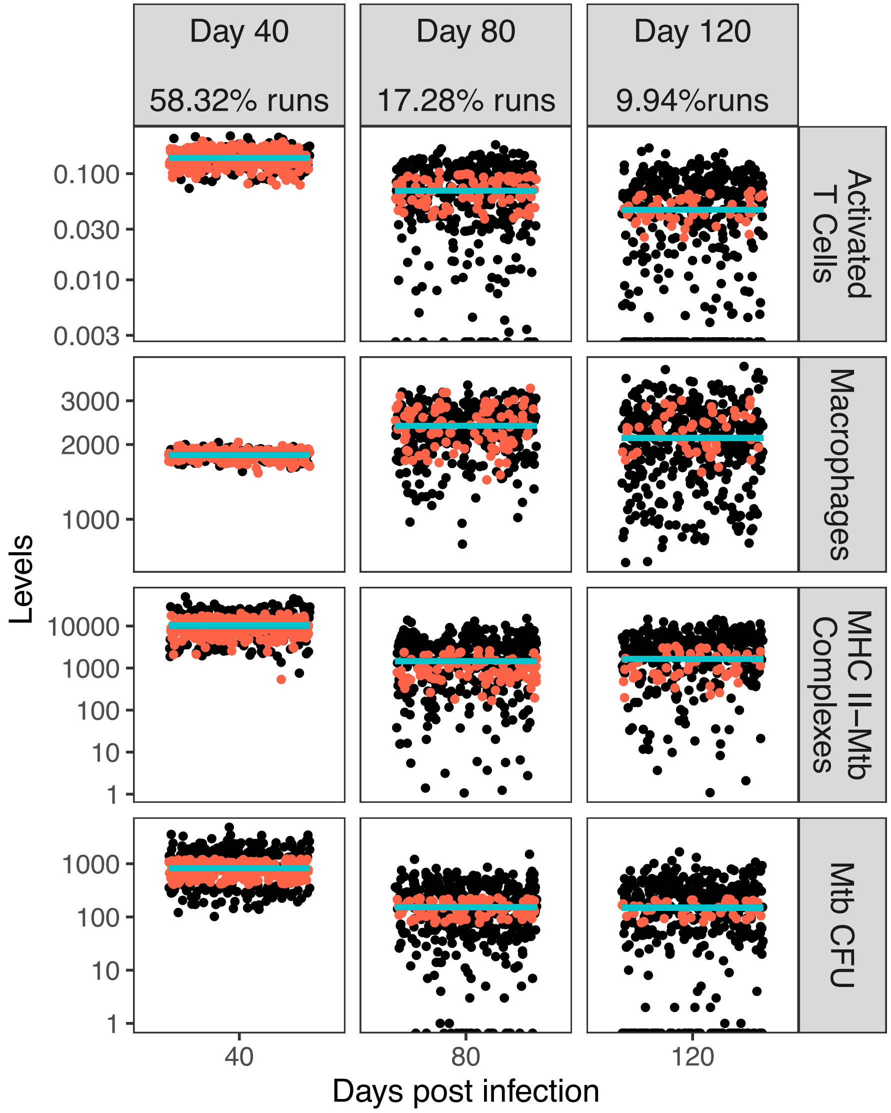

Figure S2: Chosen runs of *GranSim* simulations (red) to best representation of the median values for outputs of interest (blue). *GranSim* runs that were not chosen are shown in black. Percentage of runs chosen is shown at the top of each column. For each 4 outputs, each of the red dots fit within a +/- 50% window of the median blue line and are chosen as good representations of all runs**.**

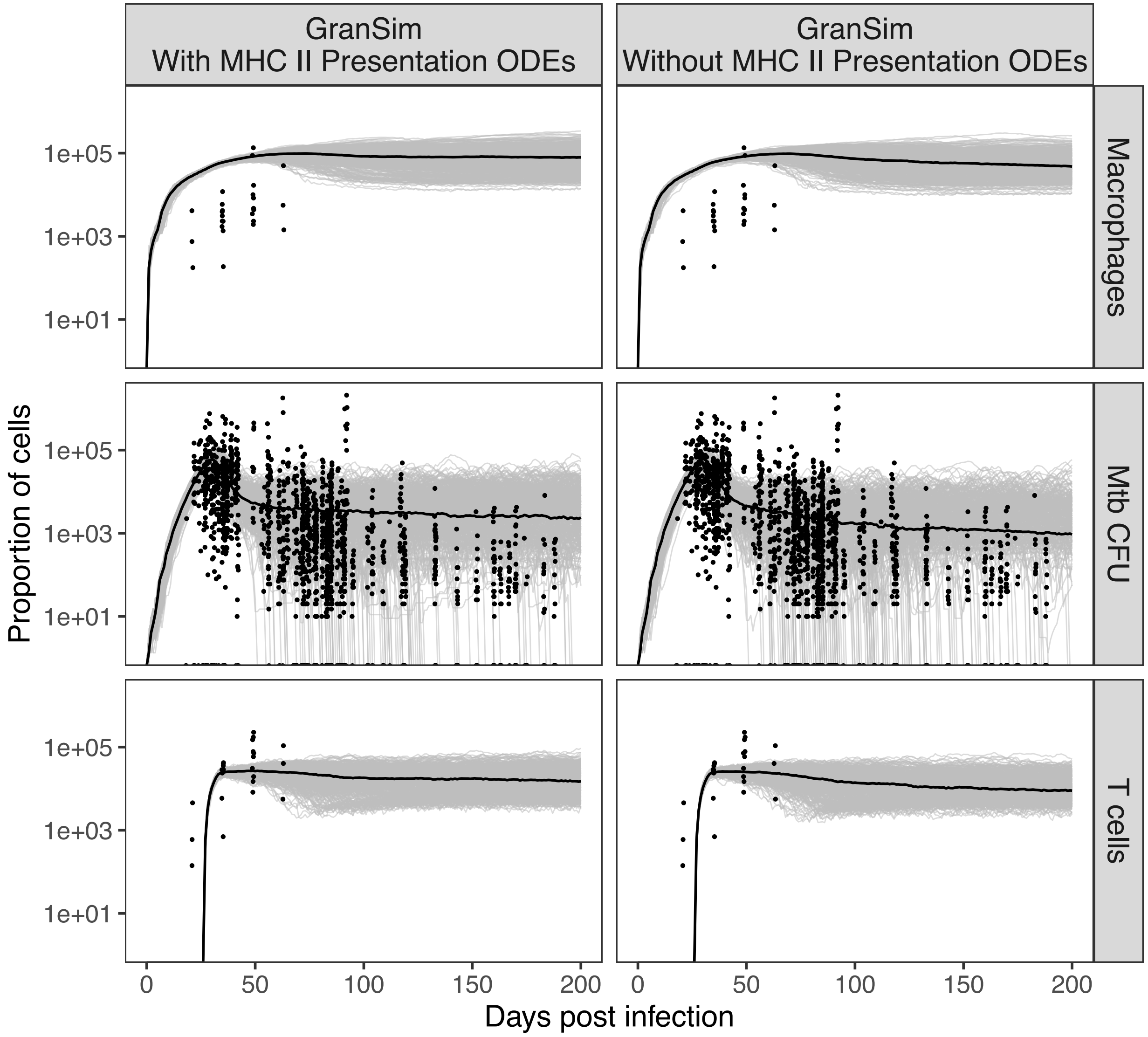

Figure S3: Comparison of *GranSim* simulations with MHCII presentation ODEs and without. Median is shown in black, with simulation runs shown in gray. NHP data is overlaid for each output (Wessler et al., 2020).

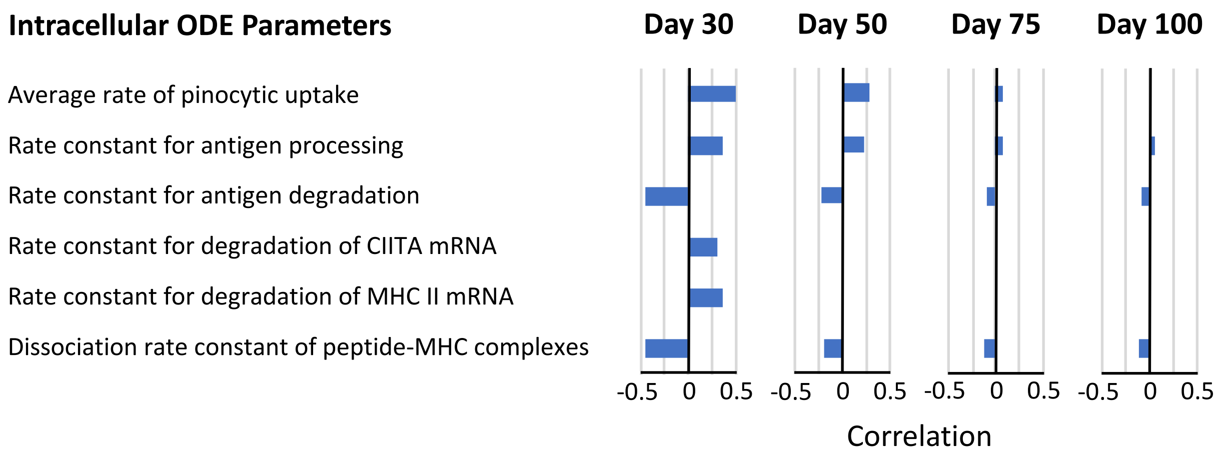

Figure S4: Results of an intercompartment sensitivity analysis. Here we fixed *GranSim* parameters and varied parameters within the intracellular scale model with a readout in the *GranSim* tissue scale model. Partial rank correlation coefficients (see Methods) for four time points are shown for six model parameters inputs with their effects on numbers of Mtb-peptide-MHCII complexes on the surface of macrophages as the output variable.

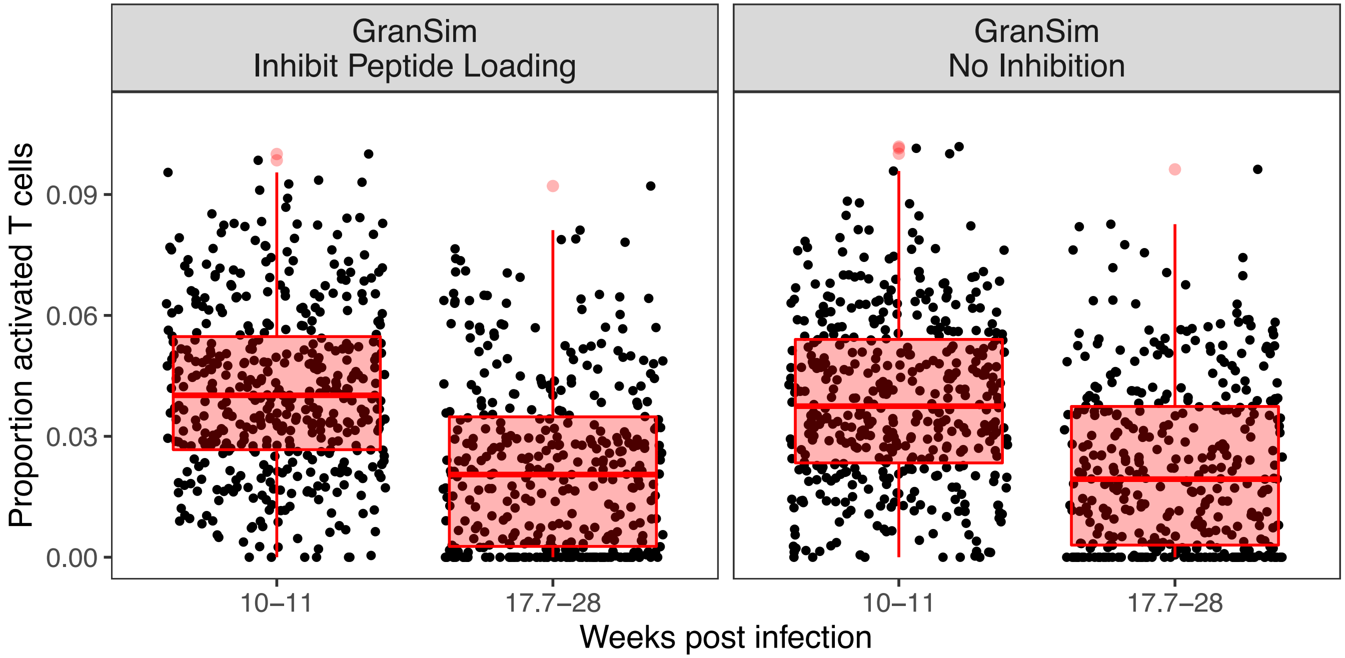

Figure S5: Proportion of activated T cells in a simulated granulomas in *GranSim* run with MHC II dynamics, comparing inhibition of peptide loading through MHCII downregulation and the negative control of no inhibition over the 28 weeks course of infection.

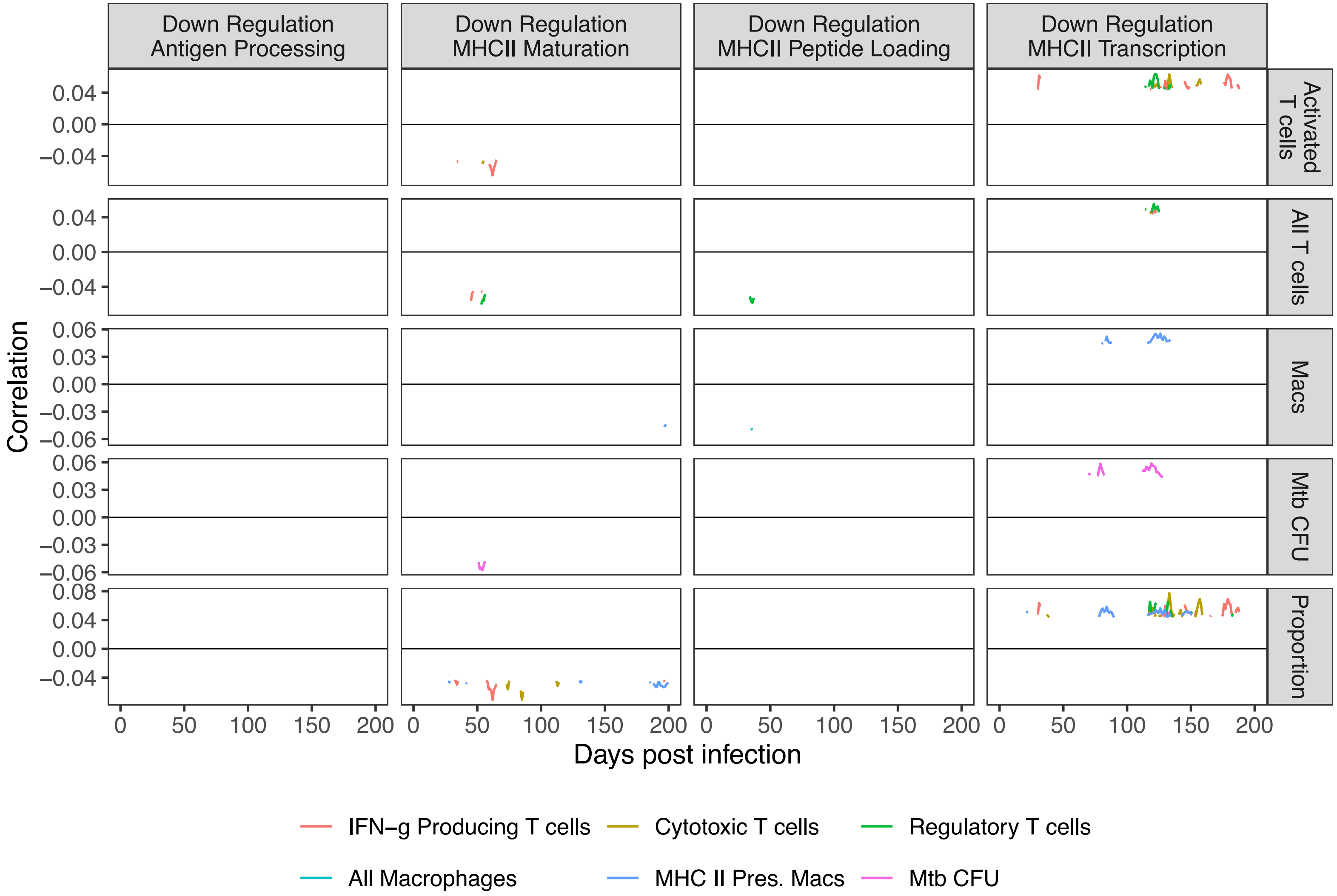

Figure S6: Results of an intra-compartment sensitivity analysis of downregulation linking parameters. Here we fixed *GranSim* and MHCII sub model parameters and varied downregulation linking parameters with a readout in the *GranSim* tissue scale model. Partial rank correlation coefficients (see Methods) for entire virtual infection are shown for the four downregulation process inputs with their effects on numbers of T cells, macrophages, and Mtb as the output variables. Only significant correlations shown ($p<0.05$).

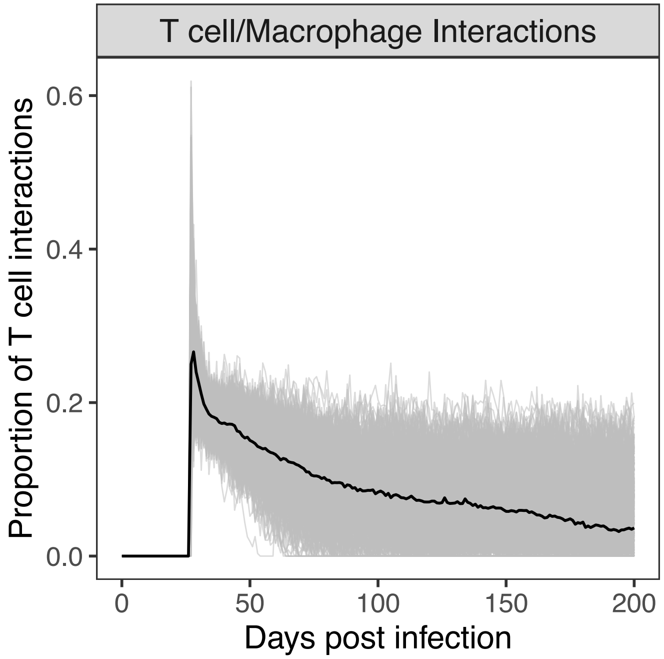

Figure S7: Proportion of T cells that interacted with at least one macrophage. Median is shown in black, with simulation runs shown in gray. *GranSim* run with MHC II dynamics, no inhibition of antigen presentation, and all other *GranSim* parameters set to default.

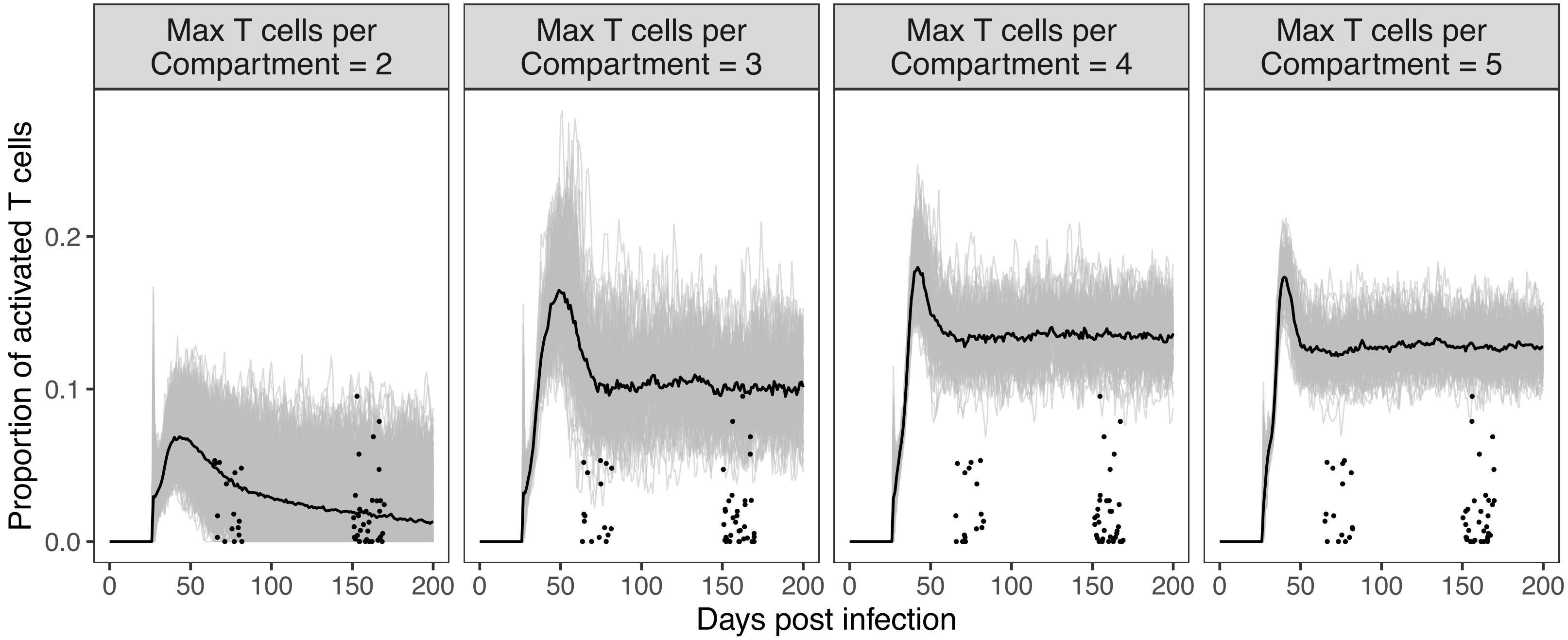

Figure S8: Comparison of proportion of activated T cells in *GranSim* simulations with MHCII presentation ODEs, varying the maximum number of T cells that can fit within one grid compartment. Median is shown in black, with simulation runs shown in gray. NHP data is overlaid and jittered over ten-day ranges (as only ranges were available) for each output (Gideon et al., 2015).

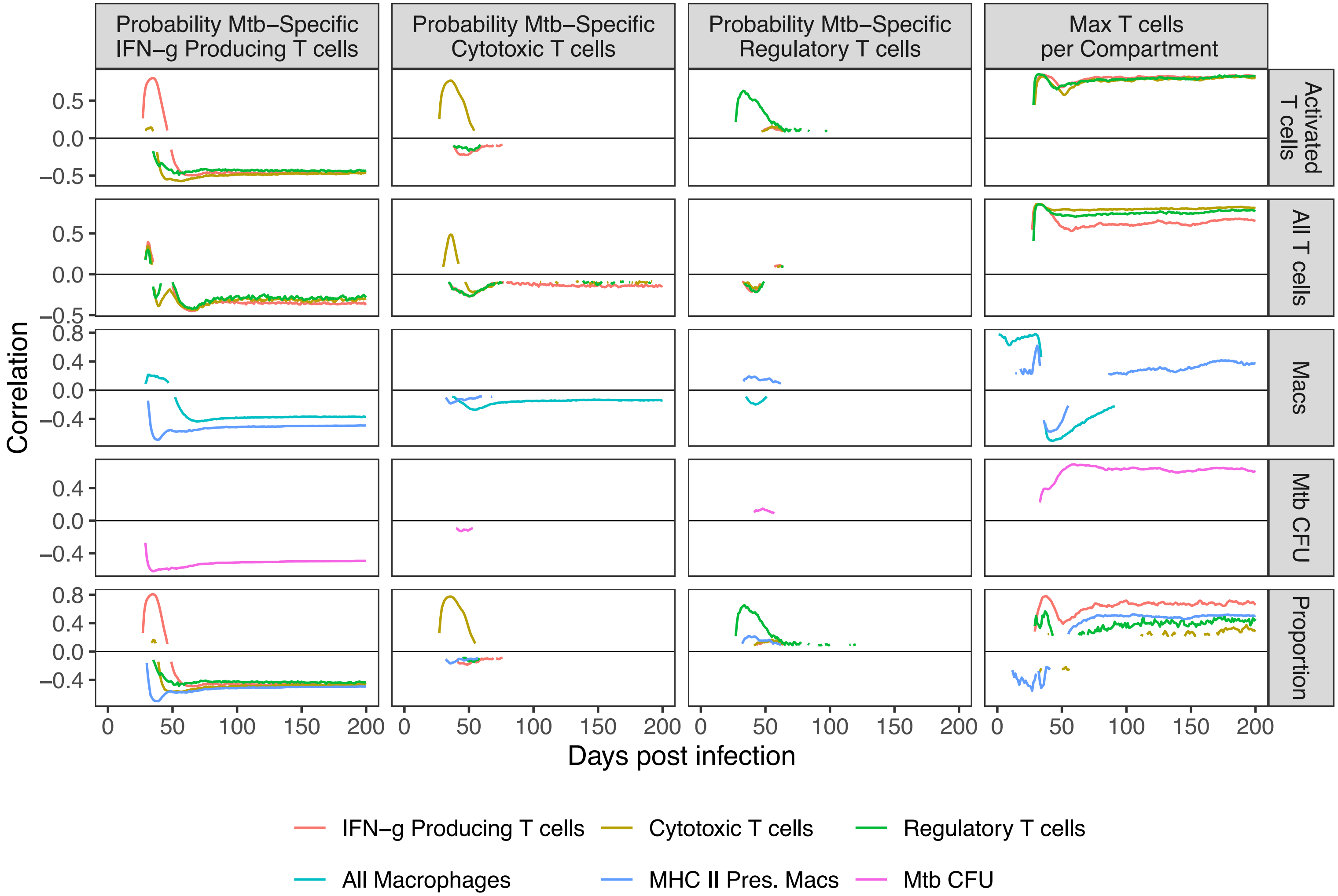

Figure S9: Results of two intra-compartment sensitivity analyses of T cell Mtb-specificity and T cell density. Here we fixed the MHCII sub model parameters and varied *GranSim* parameters for probability for T cell Mtb-specificity and separately ran and varied T cell density, with a readout in the *GranSim* tissue scale model. Partial rank correlation coefficients (see Methods) for entire virtual infection are shown for the four parameter inputs with their effects on numbers of T cells, macrophages, and Mtb as the output variables. Only significant correlations shown ($p<0.05$).

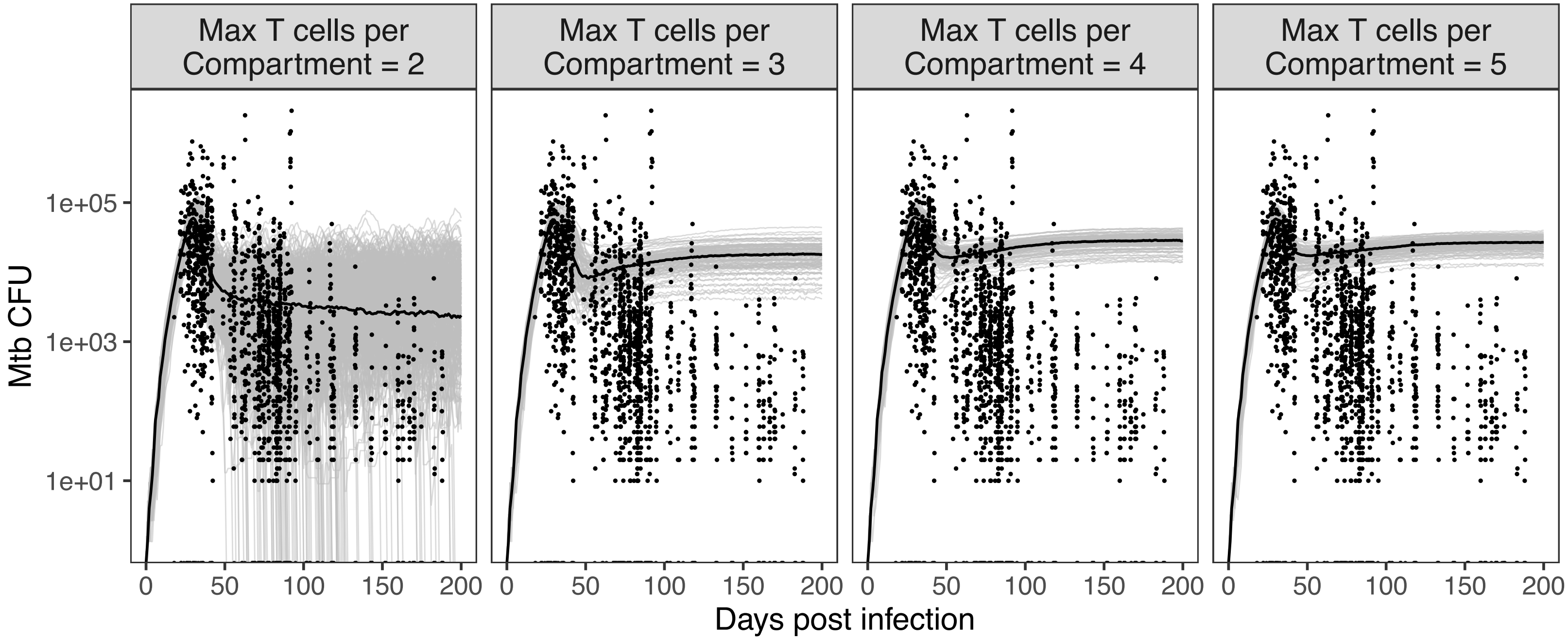

Figure S10: Comparison of Mtb clearance in *GranSim* simulations with MHCII presentation ODEs, varying the maximum number of T cells that can fit within one grid compartment. Median is shown in black, with simulation runs shown in gray. NHP data is overlaid for each output (Gideon et al., 2015).

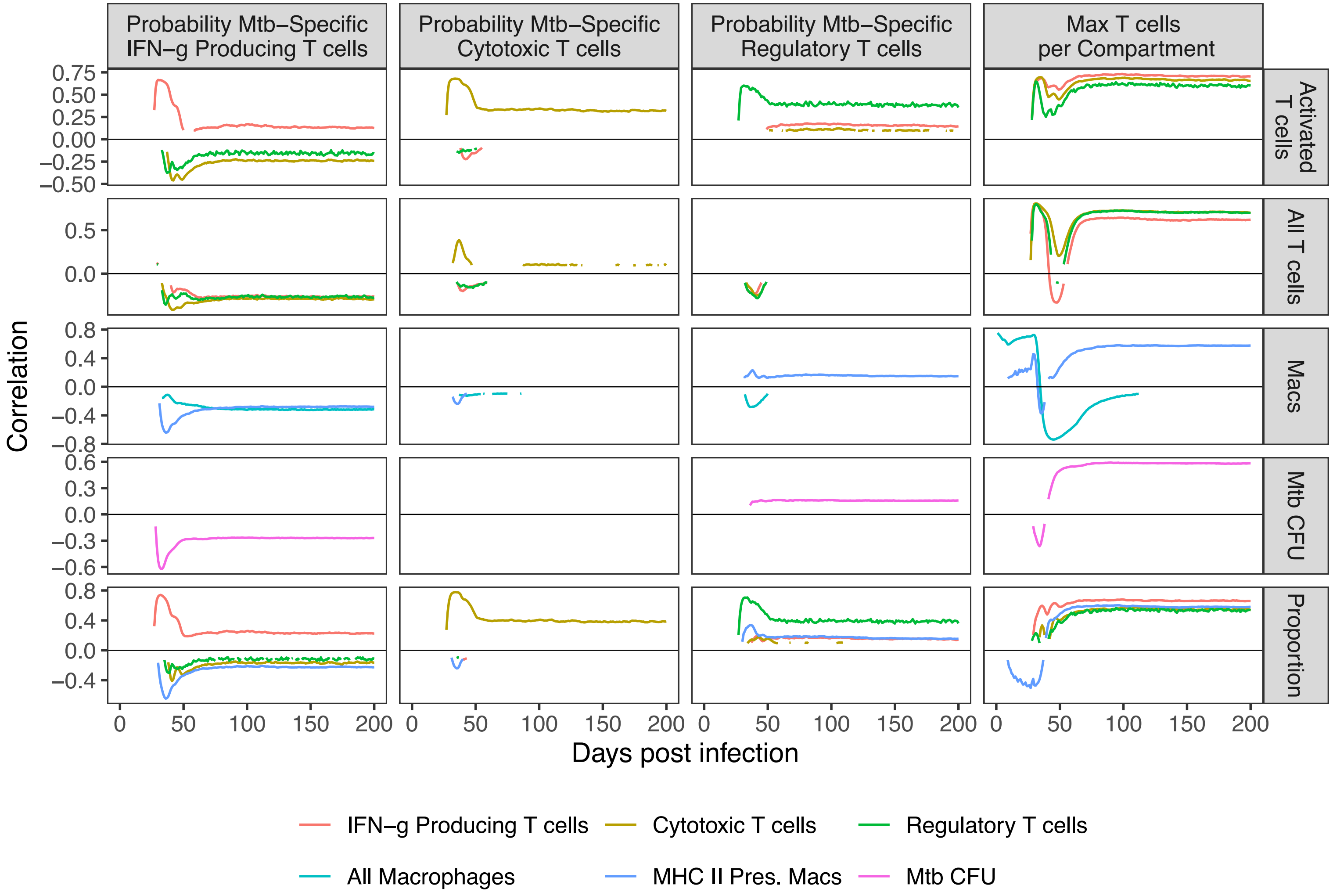

Figure S11: Results of the combined intercompartment sensitivity analysis. Here we varied both simultaneously the *GranSim* parameters and the downregulation linking parameters with a readout in the *GranSim* tissue scale model. Partial rank correlation coefficients (see Methods) for entire virtual infection are shown for the eight parameter inputs with their effects on numbers of T cells, macrophages, and Mtb as the output variables. Only significant correlations shown ($p<0.05$, columns without significant values were omitted).

Supplementary Table S1: Differential Equations Describing Molecular Scale MHCII Dynamics

Equation description	Equation
**IFN-γ Receptor Ligand Binding**	
Change in number of free IFN-γ receptors	$\frac{dR}{dt} = -k_{\text{on-IFN-}\gamma} G \cdot R + k_{\text{off-IFN-}\gamma} C + k_{\text{recyc}} C$
Change in number of IFN-γ receptor ligand complexes	$\frac{dC}{dt} = k_{\text{on-IFN-}\gamma} G \cdot R - k_{\text{off-IFN-}\gamma} C - k_{\text{recyc}} C$
**MHC Class II Transcription**	
Change in levels of CIITA mRNA	$\frac{dT_1}{dt} = k_{\text{txn1}} \left(1 + \alpha \frac{C}{R_{\text{tot}}}\right) - k_{\text{deg-mRNA1}} T_1$
Change in levels of CIITA protein	$\frac{dP}{dt} = k_{\text{tsl1}} T_1 - k_{\text{deg-P}} P$
Change in levels of MHCII mRNA	$\frac{dT_2}{dt} = k_{\text{txn2}} P - k_{\text{deg-mRNA2}} T_2$
**Mtb Antigens/Peptides and Self-Peptides**	
Change in molar concentration of Mtb antigens in endosomal compartments	$\frac{dA}{dt} = \left(k_{\text{pino}} \frac{1}{v_{\text{MIIC}}}\right) A^* - k_{\text{deg-A}} A - k_{\text{lys}} A$
Change in molar concentration of Mtb antigen-derived peptides in endosomal compartments	$\frac{dE}{dt} = k_{\text{deg-A}} A + (k_{\text{off-MHC}} M_e - k_{\text{on-MHC}} M \cdot E) \cdot \frac{1}{N_A v_{\text{MIIC}}} - k_{\text{lys}} E$
Change in molar concentration of self-peptides in endosomal compartments	$\frac{dS}{dt} = k_{\text{source}} + \left[k_{\text{deg-MHC}} (M_s + M_s^*) - k_{\text{on-MHC}} M \cdot S + k_{\text{off-MHC}} M_s\right] \left(\frac{1}{N_A v_{\text{MIIC}}}\right) - k_{\text{lys}} S$
**MHCII Translation & Peptide-MHCII Binding**	
Change in number of free MHCII proteins in endosomal compartments	$\frac{dM}{dt} = k_{\text{ts12}} + \left(1 + \beta \frac{C}{R_{\text{tot}}}\right) T - k_{\text{on-MHC}} M \cdot S + k_{\text{off-MHC}} M_s - k_{\text{on-MHC}} M \cdot E + k_{\text{off-MHC}} M_e - k_{\text{out}} M + k_{\text{in}} M^* - k_{\text{deg-MHC}} M$
Change in number of free MHCII proteins on cell surface	$\frac{dM^*}{dt} = k_{\text{out}} M - k_{\text{in}} M^* - k_{\text{deg-MHC}} M$
Change in number of MHCII self-peptide complexes in endosomal compartments	$\frac{dM_s}{dt} = k_{\text{on-MHC}} M \cdot S - k_{\text{off-MHC}} M_s - k_{\text{out}} M_s + k_{\text{in}} M_s^* - k_{\text{deg-MHC}} M_s$
Change in number of surface MHCII self-peptide complexes	$\frac{dM_s^*}{dt} = k_{\text{out}} M_s - k_{\text{in}} M_s^* - k_{\text{deg-MHC}} M_s^*$
Change in number of MHCII Mtb-peptide complexes in endosomal compartments	$\frac{dM_e}{dt} = k_{\text{on-MHC}} M \cdot P - k_{\text{off-MHC}} M_e - k_{\text{out}} M_e + k_{\text{in}} M_e^* - k_{\text{deg-MHC}} M_e$
Change in number of surface MHCII Mtb-peptide complexes	$\frac{dM_e^*}{dt} = k_{\text{out}} M_e - k_{\text{in}} M_e^* - k_{\text{deg-MHC}} M_e^*$

Supplementary Table S2: Molecular Scale MHCII Dynamics Parameters (Estimated from Chang et al., 2005)

**Parameter**	**Parameter description**	**Value (LHS Ranges)**	
$k_{\text{on-IFN-}\gamma}$	Association rate constant of IFN-γ receptor-ligand complex	$1.2 \times 10^{7}/\text{s}$,	$(5.0 \times 10^{6}, 1.3 \times 10^{7})$
$k_{\text{off-IFN-}\gamma}$	Dissociation rate constant of IFN-γ receptor-ligand complex	$9.8 \times 10^{-3}/\text{s}$,	$(2.0 \times 10^{-3}, 1.0 \times 10^{-2})$
k_{recyc}	Rate constant for receptor internalization and recycling	$2.5 \times 10^{-4}/\text{s}$,	$(1.4 \times 10^{-5}, 6.0 \times 10^{-4})$
α	Scaling factor for CIITA transcription	4.5×10^{1},	$(1.0 \times 10^{1}, 5.0 \times 10^{1})$
$k_{\text{deg-mRNA1}}$	Rate constant for degradation of CIITA mRNA	$2.1 \times 10^{-5}/\text{s}$,	$(7.0 \times 10^{-7}, 7.0 \times 10^{-5})$
$k_{\text{deg-P}}$	Rate constant for degradation of CIITA protein	$5.5 \times 10^{-4}/\text{s}$,	$(2.0 \times 10^{-5}, 8.0 \times 10^{-4})$
$k_{\text{deg-mRNA2}}$	Rate constant for degradation of MHCII mRNA	$1.7 \times 10^{-5}/\text{s}$,	$(2.0 \times 10^{-6}, 1.8 \times 10^{-5})$
k_{pino}	Average rate of pinocytic uptake per cell	$6.1 \times 10^{-17}\text{L/s}$,	$(1.4 \times 10^{-17}, 6.0 \times 10^{-16})$
$k_{\text{deg-A}}$	Rate constant for antigen processing	$1.6 \times 10^{-3}/\text{s}$,	$(6.4 \times 10^{-5}, 2.4 \times 10^{-3})$
k_{lys}	Rate constant for antigen degradation	$1.3 \times 10^{-3}/\text{s}$,	$(3.0 \times 10^{-5}, 3.0 \times 10^{-3})$
$k_{\text{on-MHC}}$	Association rate constant of peptide-MHC complexes	$1.1 \times 10^{2}/\text{M} \cdot \text{s}$,	$(5.6 \times 10^{0}, 5.6 \times 10^{3})$
$k_{\text{off-MHC}}$	Dissociation rate constant of peptide-MHC complexes	$1.3 \times 10^{-2}/\text{s}$,	$(8.0 \times 10^{-4}, 4.0 \times 10^{-2})$
$k_{\text{deg-MHC}}$	Rate constant for degradation of peptide-MHC complexes	$9.3 \times 10^{-6}/\text{s}$,	$(5.0 \times 10^{-6}, 1.3 \times 10^{-5})$
β	Scaling factor for MHCII translation	4.2×10^{0},	$(4.0 \times 10^{0}, 8.0 \times 10^{0})$
k_{out}	Rate constant of MHCII protein transport from endosomes to the plasma membrane	$4.0 \times 10^{-4}/\text{s}$,	$(6.0 \times 10^{-5}, 3.0 \times 10^{-3})$
k_{source}	Rate of self-peptide synthesis	$k_{\text{lys}} \cdot S_0$	
k_{txn1}	Rate constant for CIITA transcription	$k_{\text{deg-mRNA1}} \cdot T_{1,0}$	
k_{tsl1}	Rate constant for CIITA translation	$k_{\text{deg-P}} \cdot \frac{P_0}{T_{1,0}}$	
k_{txn2}	Rate constant for MHCII transcription	$k_{\text{deg-mRNA2}} \cdot \frac{T_{2,0}}{P_0}$	
k_{tsl2}	Rate constant representing MHCII translation	$k_{\text{deg-MHC}} \cdot \left(\frac{M_0 + M_0^* + M_{S,0} + M_{S,0}^*}{T_{2,0}}\right)$	
k_{in}	Rate constant of MHCII protein internalization from the plasma membrane	$\frac{p_{\text{in}}}{1-p_{\text{in}}} k_{\text{out}} - k_{\text{deg-MHC}}$	
v_{MIIC}	Total volume of the MHCII-accessible endosomal compartments	$4.0 \times 10^{-16}\text{L}$	
R_{tot}	IFN-γ receptors per cell	1.0×10^{4}	
p_{in}	Fraction of all MHCII that are intracellular	3.3×10^{-1}	

p_{bound}	Fraction of all MHCII that are bound to self-peptide	8.0×10^{-1}
M_{tot}	Total number of MHCII proteins on and in a macrophage	2.0×10^{5}
N_A	Avogadro's number	6.02×10^{23}

Supplementary Table S3: Molecular Scale MHCII Dynamics Variable Initial Conditions (Estimated from Chang et al., 2005)

**Variable**	**Variable description**	**Value**
R_0	Number of free IFN- γ receptors	R_{tot}
C_0	Number of IFN- γ receptor ligand complexes	0
$T_{1,0}$	Levels of CIITA mRNA	1
P_0	Levels of CIITA protein	1
$T_{2,0}$	Number of MHCII mRNA	1
A_0	Molar conc. of Mtb antigens in endosomal compartments	0
E_0	Molar conc. of Mtb peptides in endosomal compartments	0
S_0	Molar conc. of self-peptides in endosomal compartments	$\frac{k_{\text{deg-MHC}}\cdot(M_{s,0}+M_{s,0}^{*})+k_{\text{off-MHC}}\cdot M_{s,0}}{k_{\text{on-MHC}}\cdot M_0}$
M_0	Number of MHCII proteins in endosomal compartments	$p_{\text{in}}\cdot(1-p_{\text{bound}})\cdot M_{\text{tot}}$
M_0^*	Number of MHCII proteins on cell surface	$\frac{1-p_{\text{in}}}{p_{\text{in}}}\cdot M_0$
$M_{s,0}$	Number of MHCII self-peptide complexes in endosomal compartments	$\frac{p_{\text{bound}}}{1-p_{\text{bound}}}\cdot M_0$
$M_{s,0}^*$	Number of MHCII self-peptide complexes on cell surface	$\frac{1-p_{\text{in}}}{p_{\text{in}}}\cdot M_{s,0}$
$M_{e,0}$	Number of MHCII Mtb-peptide complexes in endosomal compartments	0
$M_{e,0}^*$	Number of MHCII Mtb-peptide complexes on cell surface	0

Supplementary Table S4: Molecular Scale MHCII Dynamics Scaling Factors Used to Represent Mtb Down Regulation of MHCII Processes

Parameter	Parameter description	Value
$d_{\mathrm{deg-A}}$	Maximum rate of Mtb down regulation of antigen processing ($k_{\mathrm{deg-A}}$) achieved by the system, at saturating Mtb concentration	$0.25-1.00$
d_{ts12}	Maximum rate of Mtb down regulation of MHCII maturation (k_{ts12}) achieved by the system, at saturating Mtb concentration	$0.25-1.00$
$d_{\mathrm{on-MHC}}$	Maximum rate of Mtb down regulation of MHCII peptide loading ($k_{\mathrm{on-MHC}}$) achieved by the system, at saturating bacterial concentration	$0.25-1.00$
d_{txn1}	Maximum rate of Mtb down regulation of MHCII transcription (k_{txn1}) achieved by the system, at saturating Mtb concentration	$0.25-1.00$
d_{half}	Number of intracellular Mtb at which the reaction rate is half of Mtb down regulation	5

Supplementary Table S5: Mtb Down Regulation of MHCII Processes Equations

Equation description	Equation
Mtb down regulation of antigen processing	$\mathrm{mtbDownReg}_{\mathrm{deg-A}} = 1 - \frac{d_{\mathrm{deg-A}} \cdot (\#\ \mathrm{Mtb\ in\ Mac})}{d_{\mathrm{half}} + (\#\ \mathrm{Mtb\ in\ Mac})}$
Mtb down regulation of MHCII maturation	$\mathrm{mtbDownReg}_{\mathrm{ts12}} = 1 - \frac{d_{\mathrm{ts12}} \cdot (\#\ \mathrm{Mtb\ in\ Mac})}{d_{\mathrm{half}} + (\#\ \mathrm{Mtb\ in\ Mac})}$
Mtb down regulation of MHCII peptide loading	$\mathrm{mtbDownReg}_{\mathrm{on-MHC}} = 1 - \frac{d_{\mathrm{on-MHC}} \cdot (\#\ \mathrm{Mtb\ in\ Mac})}{d_{\mathrm{half}} + (\#\ \mathrm{Mtb\ in\ Mac})}$
Mtb down regulation of MHCII transcription	$\mathrm{mtbDownReg}_{\mathrm{txn1}} = 1 - \frac{d_{\mathrm{txn1}} \cdot (\#\ \mathrm{Mtb\ in\ Mac})}{d_{\mathrm{half}} + (\#\ \mathrm{Mtb\ in\ Mac})}$
